# Macrophage RAGE activation is proinflammatory in NASH

**DOI:** 10.1172/jci.insight.169138

**Published:** 2024-02-08

**Authors:** Gopanandan Parthasarathy, Amy S. Mauer, Naresh Golla, P. Vineeth Daniel, Lily H. Kim, Guneet S. Sidhu, George W. Marek, Emilien Loeuillard, Anuradha Krishnan, Hyun Se Kim Lee, Kevin D. Pavelko, Michael Charlton, Petra Hirsova, Sumera I. Ilyas, Harmeet Malhi

**Affiliations:** 1 Division of Gastroenterology and Hepatology, Mayo Clinic, Rochester, Minnesota, USA.; 2 Department of Biochemistry, UT Southwestern Medical Center, Dallas, Texas, USA.; 3 Department of Internal Medicine, University of North Dakota, Fargo, North Dakota, USA.; 4 Department of Immunology, Mayo Clinic, Rochester, Minnesota.; 5 Section of Gastroenterology, Hepatology and Nutrition, Department of Medicine, The University of Chicago, Chicago, Illinois, USA.

**Keywords:** Hepatology, Inflammation, Drug therapy, Hepatitis, Macrophages

## Abstract

Intrahepatic macrophages in nonalcoholic steatohepatitis (NASH) are heterogenous and include proinflammatory recruited monocyte-derived macrophages. The receptor for advanced glycation endproducts (RAGE) is expressed on macrophages and can be activated by damage associated molecular patterns (DAMPs) upregulated in NASH, yet the role of macrophage-specific RAGE signaling in NASH is unclear. Therefore, we hypothesized that RAGE-expressing macrophages are proinflammatory and mediate liver inflammation in NASH. Compared with healthy controls, RAGE expression was increased in liver biopsies from patients with NASH. In a high-fat, -fructose, and -cholesterol–induced (FFC)-induced murine model of NASH, RAGE expression was increased, specifically on recruited macrophages. FFC mice that received a pharmacological inhibitor of RAGE (TTP488), and myeloid-specific RAGE KO mice (RAGE-MKO) had attenuated liver injury associated with a reduced accumulation of RAGE^+^ recruited macrophages. Transcriptomics analysis suggested that pathways of macrophage and T cell activation were upregulated by FFC diet, inhibited by TTP488 treatment, and reduced in RAGE-MKO mice. Correspondingly, the secretome of ligand-stimulated BM-derived macrophages from RAGE-MKO mice had an attenuated capacity to activate CD8^+^ T cells. Our data implicate RAGE as what we propose to be a novel and potentially targetable mediator of the proinflammatory signaling of recruited macrophages in NASH.

## Abstract 2

<p>RAGE is upregulated in human and murine NASH on recruited macrophages in the liver and can be targeted to ameliorate&nbsp;liver injury via&nbsp;reduction of&nbsp;proinflammatory macrophage activation.</p>

## Graphical abstract

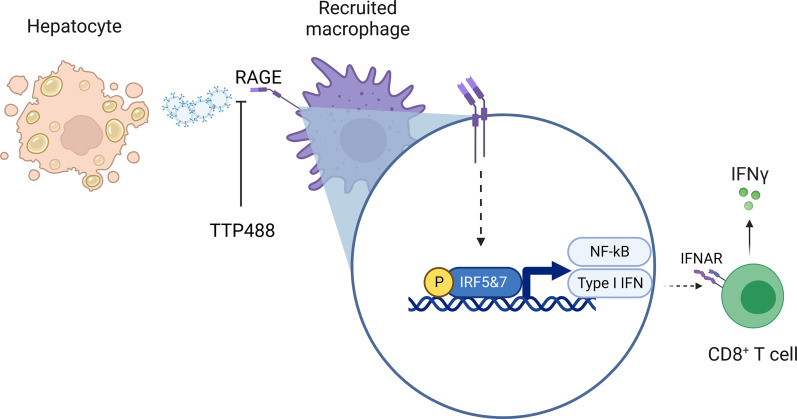

## Introduction

Nonalcoholic fatty liver disease (NAFLD) is the most common chronic liver disease in the United States, and its pathogenesis is complex (1). The insult of hepatocyte lipotoxicity is accompanied by contributions from immune system activation and modifiers such as genetic susceptibility, alcohol use, and intestinal dysbiosis (2). Macrophages are the most abundant immune cell type in the liver and are crucially implicated in the initiation and propagation of sterile inflammation and the regulation of fibrosis in NASH (3). Liver macrophages are a heterogeneous population consisting of resident macrophages that are yolk sac derived, historically termed Kupffer cells (KCs); and are recruited or monocyte-derived macrophages that originate from BM precursors. Several recent studies have described transcriptomics-based subsets of macrophages in both mouse models and human NASH (4–7). However, the functional relevance of macrophage subsets and the precise cellular signaling pathways that regulate them are incompletely elucidated. Furthermore, while macrophages are known to be activated by several proinflammatory ligands, the receptors engaged by these ligands and receptor-mediated effector functions remain partially defined.

The receptor for advanced glycation endproducts (RAGE), a member of the immunoglobulin superfamily, serves as a multiligand pattern-recognition receptor that can bind several ligands such as AGEs that accumulate in hyperglycemia as well as various damage-associated molecular patterns (DAMPs) in sterile inflammation such as S100 proteins (8). RAGE is expressed on monocytes and macrophages, where its expression is upregulated in sterile inflammatory conditions (9). For example, under conditions of chronic inflammation, such as diabetic vasculopathy and atherosclerosis, RAGE signaling is strikingly upregulated on macrophages, mediating and amplifying proinflammatory responses. Some experimental models employing whole-body deletion of RAGE have described a proinflammatory role for RAGE-mediated signaling in diet-induced murine NASH (10), while others have shown conflicting results (11, 12), suggesting that the RAGE signaling may vary by cell type. For instance, a recent study demonstrated that hepatocyte-RAGE signaling mediates liver inflammation and fibrosis in mice fed a diet high in AGEs (13). However, the cell-specific role of RAGE signaling in macrophage-mediated inflammation in NASH is less well defined.

Therefore, our objective was to investigate the role of macrophage RAGE signaling in NASH pathogenesis. Due to promiscuity of ligands that can activate RAGE, we focused instead on RAGE inhibition and deletion to demonstrate that RAGE is induced in NASH on a subset of recruited monocyte-derived macrophages in response to diet-induced liver injury. RAGE upregulation is associated with induction of proinflammatory type I IFN signaling. A pharmacological small-molecule inhibitor of RAGE attenuates macrophage-mediated inflammation and offers a potential therapeutic target in NASH. Finally, myeloid-specific genetic deletion of RAGE also attenuates proinflammatory signaling and crosstalk with CD8^+^ T cells and, thus, may attenuate NASH.

## Results

### RAGE is upregulated in murine and human NASH.

**Figure 1:**
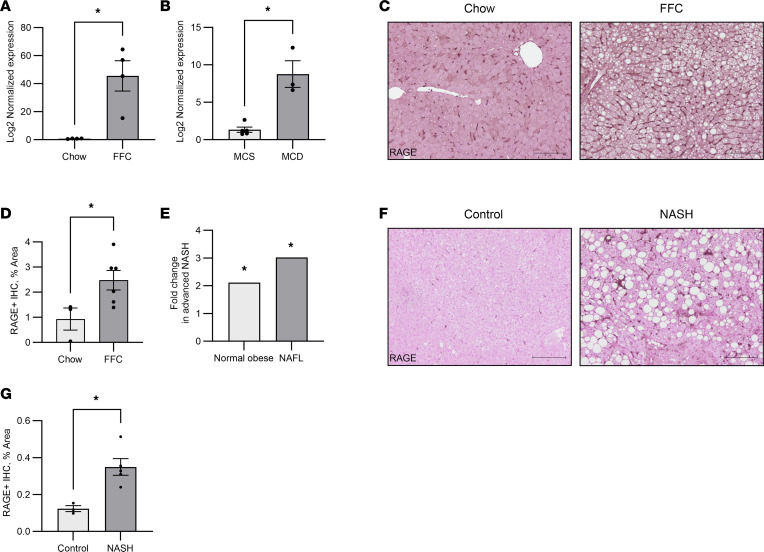
RAGE expression is upregulated in human and murine NASH. (**A**) Relative mRNA expression of *Ager* in FFC- compared with chow-fed mice (*n* = 4 each), *P* < 0.05. (**B**) MCD- (*n* = 3) compared with MCS-fed mice (*n* = 5), *P* < 0.05. (**C**) Representative images of RAGE IHC from FFC (*n* = 6) and chow (*n* = 3) mice. Scale bar: 50 μm. (**D**) Quantification of **C**; *P* < 0.05. (**E**) Fold-change of mRNA expression of *AGER* in whole-liver RNA-Seq of biopsies obtained from patients with advanced NASH on the *y* axis (*n* = 12) relative to 2 groups on the *x* axis — normal obese (*n* = 9, *P* < 0.05) and simple steatosis (*n* = 10, *P* < 0.05). (**F**) Representative images of RAGE IHC from patients with NASH (*n* = 5) and healthy patients (*n* = 3). Scale bar: 50 μm. (**G**) Quantification from **F**; *P* < 0.05. Mann-Whitney *U* test was used for statistical analyses,

To confirm prior reports of RAGE upregulation in NASH, the mRNA and protein abundance of RAGE was interrogated in 2 dietary murine models of NASH and in human NASH liver samples. Two separate murine models of diet-induced NASH were employed in male mice — a relatively rapid model where mice were placed on the methionine- and choline-deficient (MCD) diet for 4 weeks (14) and a long-term feeding model that is more reflective of human NASH, where mice were placed on a high-fat, -fructose, and -cholesterol (FFC) diet for a total of 24 weeks (15). This time point was selected based on previous time course data that demonstrate that, at 20–24 weeks duration of FFC feeding, mice develop systemic features of obesity and insulin resistance and hepatic features of steatosis, inflammation, and fibrosis (16). The mRNA abundance of *Ager* was significantly greater in mice fed FFC compared with a chow diet (Figure 1A), and it was significantly greater in MCD compared with the control methionine- and choline-sufficient (MCS) diet (Figure 1B). Correspondingly, by IHC, RAGE expression was greater in livers of FFC mice compared with chow (Figure 1, C and D). In a cohort of patients, we examined the expression of *AGER* in whole liver using RNA-Seq of liver biopsy samples. Patients with advanced NASH had a 3-fold and 2-fold increase in *AGER* expression compared with obese normal and BMI matched controls without NASH, respectively (Figure 1E). At the protein level, liver biopsies from patients with NASH compared with healthy individuals also demonstrated an upregulation of RAGE expression based on IHC (Figure 1, F and G). Since RAGE is known to be expressed on endothelial cells, hepatocytes, and immune cells, we wanted to next clarify the cell-specific expression of RAGE upregulation in NASH.

### RAGE is enriched on recruited macrophages.

**Figure 2:**
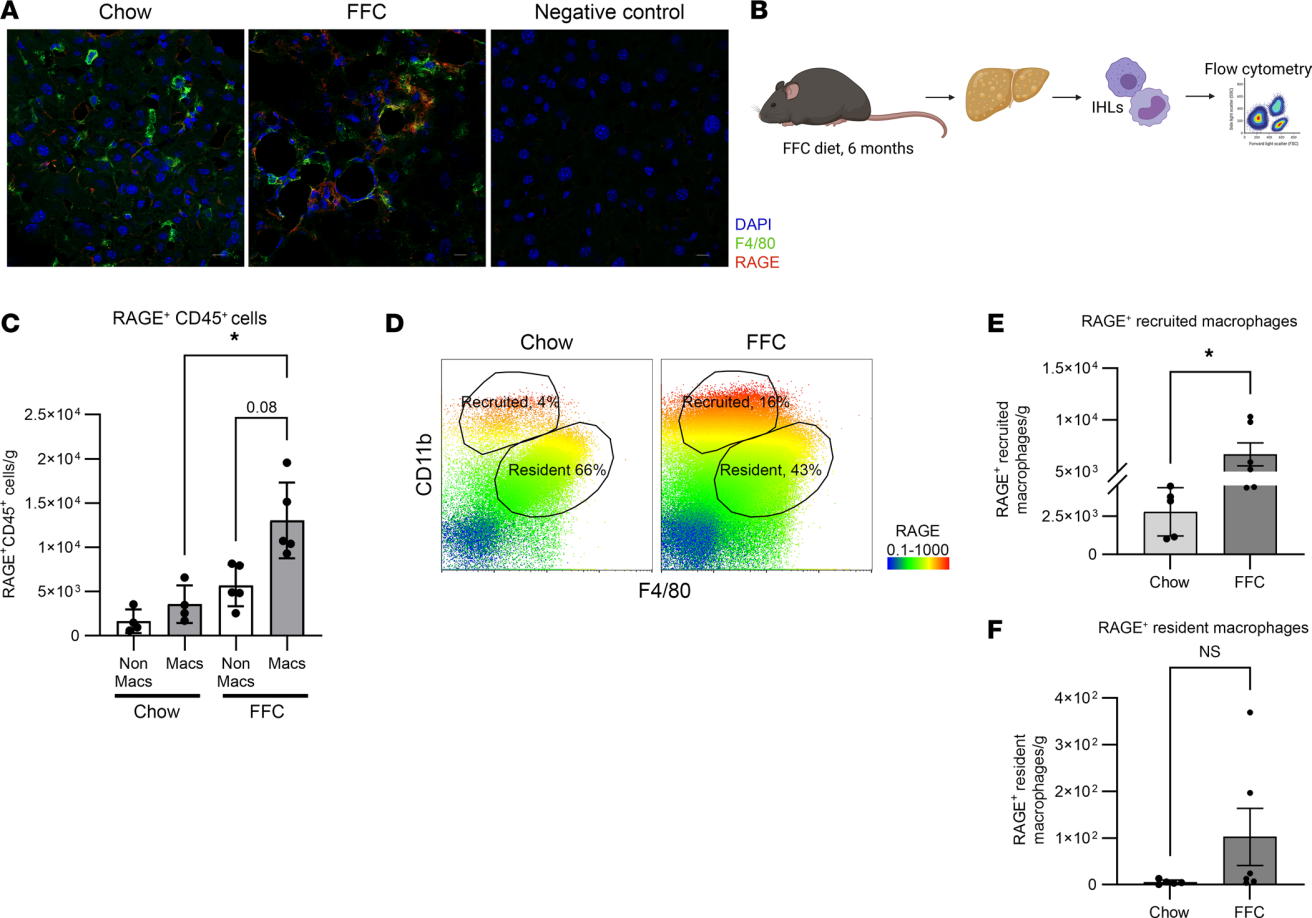
RAGE elevation in murine NASH is enriched on recruited macrophages. (**A**) Representative co-IF images of liver cryosections from FFC mice (*n* = 6) compared with chow mice (*n* = 5), stained for DAPI, F4/80, and RAGE. A negative control is shown. Scale bar: 50 μm. (**B**) Schematic depicting experimental design for isolating IHLs for flow cytometry from FFC mice. (**C**) Quantification of RAGE^+^ among F4/80^–^CD45^+^ (nonmacrophages [NonMacs]) and F4/80^+^CD45^+^ (macrophages [Macs]) cells in livers from chow and FFC mice (*n* = 4 chow, *n* = 5 FFC, *P* < 0.01). Cell numbers are presented as cell count per gram of liver. Mann-Whitney *U* test was used for statistical analyses, and *P* values were adjusted by the Benjamini-Hochberg method for multiple comparisons. (**D**) Representative flow cytometry gating demonstrating RAGE expression, depicted as a heatmap of RAGE expression intensity, among recruited (CD11b^hi^F4/80^int^) and resident macrophages (CD11b^int^F4/80^hi^) in chow and FFC mice. (**E**) Quantification of RAGE^+^ recruited macrophages in chow (*n* = 5) and FFC (*n* = 6) mice; *P* < 0.05. (**F**) Quantification of RAGE^+^ resident macrophages in chow (*n* = 5) and FFC (*n* = 6) mice; *P* < 0.05. Cell numbers are presented as cell count per gram of liver. Mann-Whitney *U* test was used for statistical analyses, and *P* values were adjusted by the Benjamini-Hochberg method for multiple comparisons.

RAGE is known to be induced in macrophages in obesity (9), and RAGE IHC in mouse and human NASH livers demonstrated RAGE positivity along hepatic sinusoids (Figure 1C), suggesting a macrophage expression pattern. We confirmed RAGE expression on macrophages by co-IHC for RAGE and a macrophage marker (CD68) on formalin-fixed paraffin-embedded human liver biopsies from healthy controls and patients with NASH (Supplemental Figure 1; supplemental material available online with this article; https://doi.org/10.1172/jci.insight.169138DS1). Additionally, we performed coimmunofluorescence (co-IF) of cryopreserved liver sections of mice fed the FFC diet for 24 weeks. Through co-IF staining for RAGE and the macrophage marker F4/80, we determined that RAGE was upregulated on macrophages within hepatic sinusoids (Figure 2A). We next interrogated RAGE expression on all intrahepatic leukocytes (IHLs) from mice fed a chow or FFC diet by flow cytometry (Figure 2B). Nonparenchymal liver cells were isolated using a combination of enzymatic digestion and Percoll density gradient centrifugation (17). From this isolation, among all live CD45^+^ cells, RAGE expression was assessed on macrophages (CD45^+^F4/80^+^) versus nonmacrophages (CD45^+^F4/80^–^). RAGE^+^ macrophages were significantly more abundant in FFC mouse livers compared with macrophages in chow mouse livers (Figure 2C). Furthermore, there were more RAGE^+^ macrophages than nonmacrophages in FFC mouse livers, while the number of RAGE^+^ macrophages and nonmacrophages were comparable in chow mouse livers, demonstrating an increase in the RAGE^+^ macrophage pool in FFC mouse livers (Figure 2C). The liver macrophage pool is broadly divided into resident and recruited macrophages, historically distinguished based on relative intensity of F4/80 and CD11b expression. Using this classification, we found that the intensity of RAGE expression was significantly greater on recruited macrophages (CD11b^hi^ F4/80^int^) compared with expression in resident macrophages (CD11b^int^ F4/80^hi^) and was increased on recruited macrophages in FFC compared with chow mice (Figure 2D). We next determined the number of RAGE^+^ recruited macrophages and found them to be significantly greater in FFC compared with chow mice, following 6 months of FFC feeding (Figure 2E), and significantly greater than RAGE^+^ resident macrophages (Figure 2F).

### Inhibition of RAGE attenuates liver injury.

**Figure 3:**
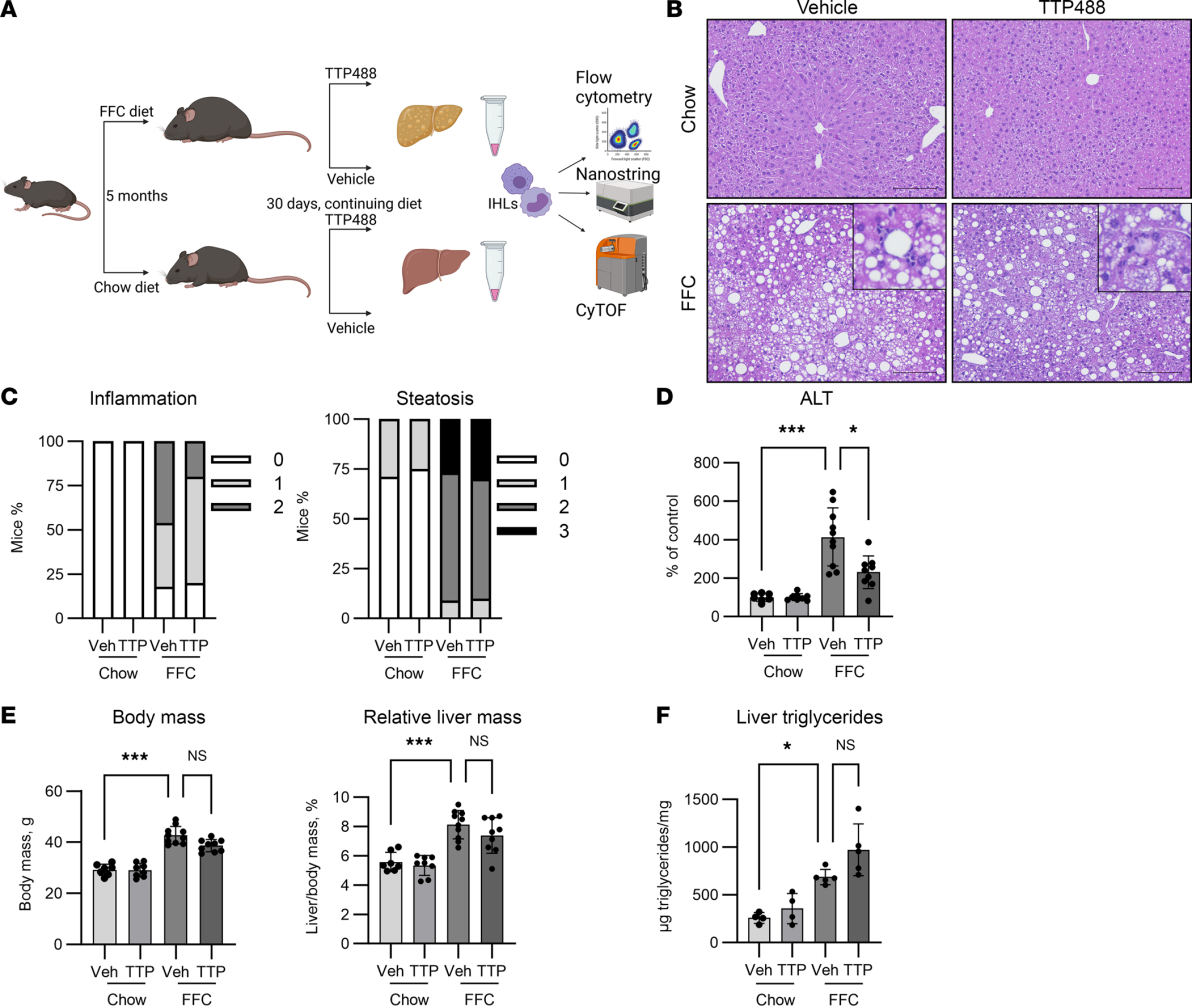
Pharmacological inhibition of RAGE attenuates diet induced NASH. (**A**) Schematic depicting experimental design for pharmacological inhibition of RAGE. (**B**) Representative images of H&E-stained livers from vehicle- or TTP488-treated chow (*n* = 7 and *n* = 8, respectively) and FFC mice (*n* = 11 and *n* = 10, respectively) demonstrating inflammatory foci (inset showing region of interest at same magnification). Scale bar: 50 μm. (**C**) Distribution of inflammation and steatosis subscores of the NAFLD activity score. (**D**) Comparison of serum ALT levels standardized to vehicle-treated chow mice (Chow-vehicle *n* = 7, Chow-TTP *n* = 8, FFC vehicle *n* = 10, FFC-TTP *n* = 9; *P* < 0.05). (**E**) Comparison of body mass (Chow-vehicle *n* = 7, Chow-TTP *n* = 8, FFC vehicle *n* = 10, FFC-TTP *n* = 9; *P* < 0.001) and relative liver mass (Chow-vehicle *n* = 7, Chow-TTP *n* = 8, FFC vehicle *n* = 10, FFC-TTP *n* = 9; *P* < 0.001). (**F**) Liver triglyceride content (Chow-vehicle *n* = 4, Chow-TTP *n* = 4, FFC vehicle *n* = 5, FFC-TTP *n* = 5; *P* < 0.05). Mann-Whitney *U* test was used for statistical analyses, and *P* values were adjusted by the Benjamini-Hochberg method for multiple comparisons.

Next, we inhibited RAGE signaling using a pharmacological inhibitor (TTP488) administered as daily i.p. injections at a dose of 4 mg/kg, reported to be efficacious for in vivo immune modulation of murine T cells (18), or equivalent volume of vehicle (10% DMSO, 40% PEG300, 5% Tween 80, and 45% ddH_2_0) for 30 days, while they continued to be on their respective diets. We administered TTP488 or vehicle to male mice with established NASH following 5 months of FFC diet feeding (Figure 3A), having confirmed an increase in RAGE-expressing recruited macrophages at this duration of feeding. Compared with chow, FFC mice had greater liver injury, which was attenuated in TTP488-treated mice, as assessed by examination of inflammatory foci on H&E (Figure 3B). Histological assessment of steatosis and inflammation according to the NAFLD activity score (NAS) demonstrated a reduction in inflammation from nearly 50% of mice with grade 2 inflammation in the vehicle-treated mice to less than 25% in the TTP488-treated mice, with no change in steatosis (Figure 3C). A reduction in plasma alanine aminotransferase (ALT), although etiologically nonspecific, was indicative of lesser liver injury in TTP488-treated mice (Figure 3D). Overall, FFC mice had greater body mass and relative liver mass (Figure 3E), but these were not significantly different between vehicle- and TTP488-treated mice. Liver triglyceride content was increased with FFC diet but unchanged with TTP488 treatment (Figure 3F). Liver fibrosis was quantified by polarized light imaging of sirius red staining (Supplemental Figure 2A). As expected, fibrosis was greater in FFC compared with chow mice and was numerically reduced in TTP488-treated mice, although not to a statistically significant degree (Supplemental Figure 2B). Furthermore, food intake after initiation of drug therapy was not different between the groups (Supplemental Figure 3A). Metabolic parameters were also similar– AUC during glucose tolerance test, relative fat and lean mass, resting energy expenditure, activity levels, respiratory exchange ratio, and heat production were all unaffected by TTP488 treatment (Supplemental Figure 3, B–H). We next examined changes in IHLs to determine if mitigation of liver injury by TTP488 was associated with changes in macrophage subsets.

### TTP488 attenuates FFC diet–induced RAGE^+^ macrophage recruitment.

**Figure 4:**
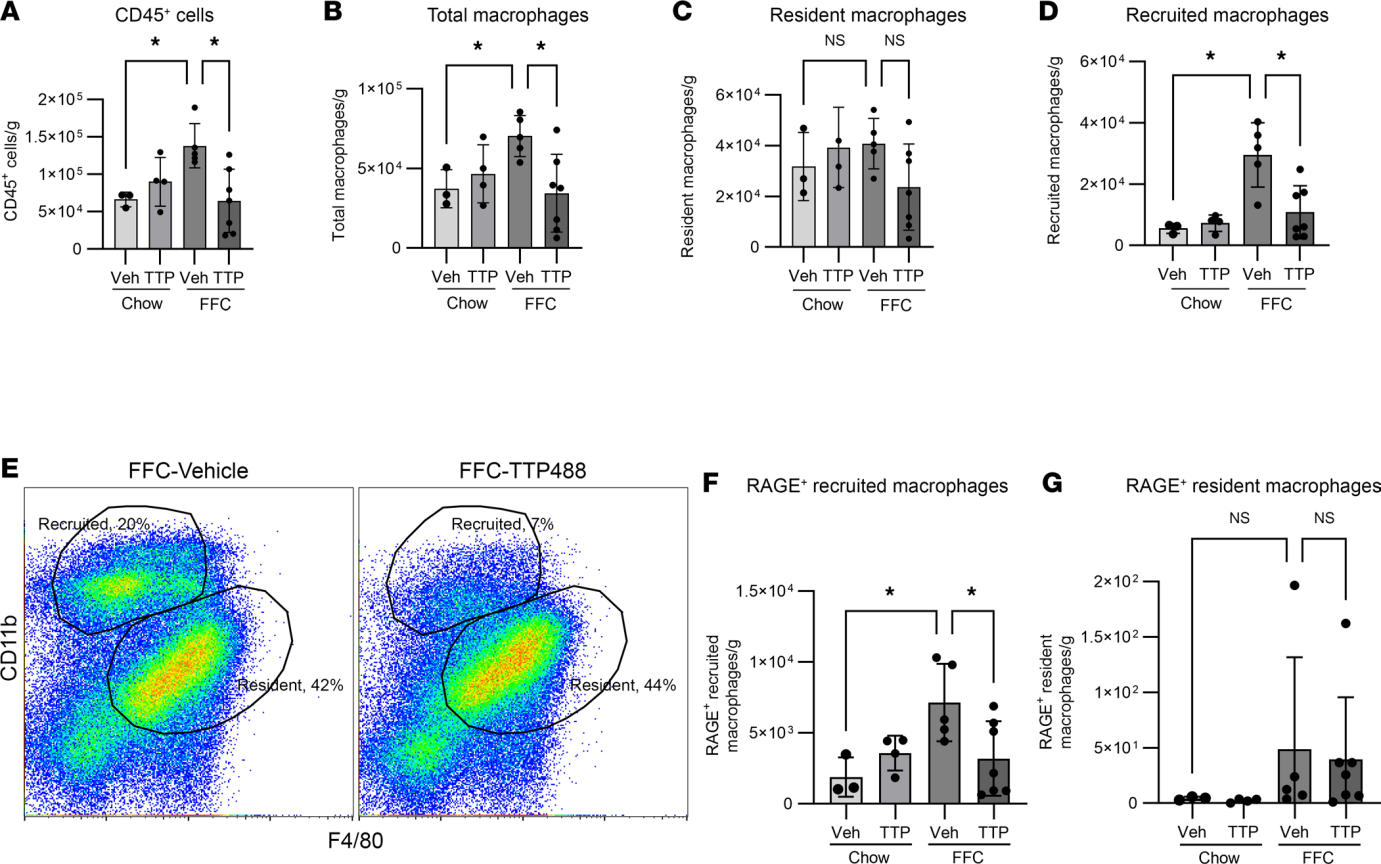
TTP488 attenuates accumulation of RAGE^+^ recruited macrophages. (**A**) Quantification of CD45^+^ cells in livers from vehicle- or TTP488-treated chow and FFC mice (Chow-vehicle *n* = 3, Chow-TTP *n* = 4, FFC vehicle *n* = 5, FFC-TTP *n* = 7; *P* < 0.05). (**B**) Total macrophages (*P* < 0.05). (**C**) Resident macrophages (CD11b^int^F4/80^hi^) (*P* = 0.06). (**D**) Recruited (CD11b^hi^ F4/80^int^) macrophages (*P* < 0.05). (**E**) Representative flow cytometry gating depicting recruited (CD11b^hi^F4/80^int^) and resident macrophages (CD11b^int^F4/80^hi^) macrophages. (**F**) Quantification of RAGE^+^ recruited macrophages (*P* < 0.05). (**G**) Quantification of RAGE^+^ resident macrophages (*P* < 0.05). Cell numbers are presented as cell count per gram of liver. Mann-Whitney *U* test was used for statistical analyses, and *P* values were adjusted by the Benjamini-Hochberg method for multiple comparisons.

By flow cytometry, and consistent with previous work, total numbers of CD45^+^ cells and macrophages were greater in FFC mice and were significantly reduced in TTP488-treated FFC mice compared with vehicle-treated mice (Figure 4, A and B). By flow cytometry, resident macrophages (CD11b^int^F4/80^hi^) were similar across diet and treatment arms (Figure 4C), but recruited macrophages (CD11b^hi^ F4/80^int^) were significantly increased in FFC compared with chow and reduced in TTP488-treated FFC mice (Figure 4, D and E). Subset analysis demonstrated that the abundance of RAGE^+^ recruited macrophages (Figure 4F) was greater in FFC compared with chow and was reduced in TTP488-compared with vehicle-treated FFC mice. In contrast, RAGE^+^ resident macrophages were few in number, representing only approximately 1% of all resident macrophages, and their abundance was not different between FFC and chow mice (Figure 4G). Thus, RAGE^+^ recruited macrophages were increased in diet-induced NASH, and their intrahepatic accumulation was attenuated by pharmacological RAGE inhibition.

### FFC diet–induced macrophage and T cell activation is attenuated by RAGE inhibition.

**Figure 5:**
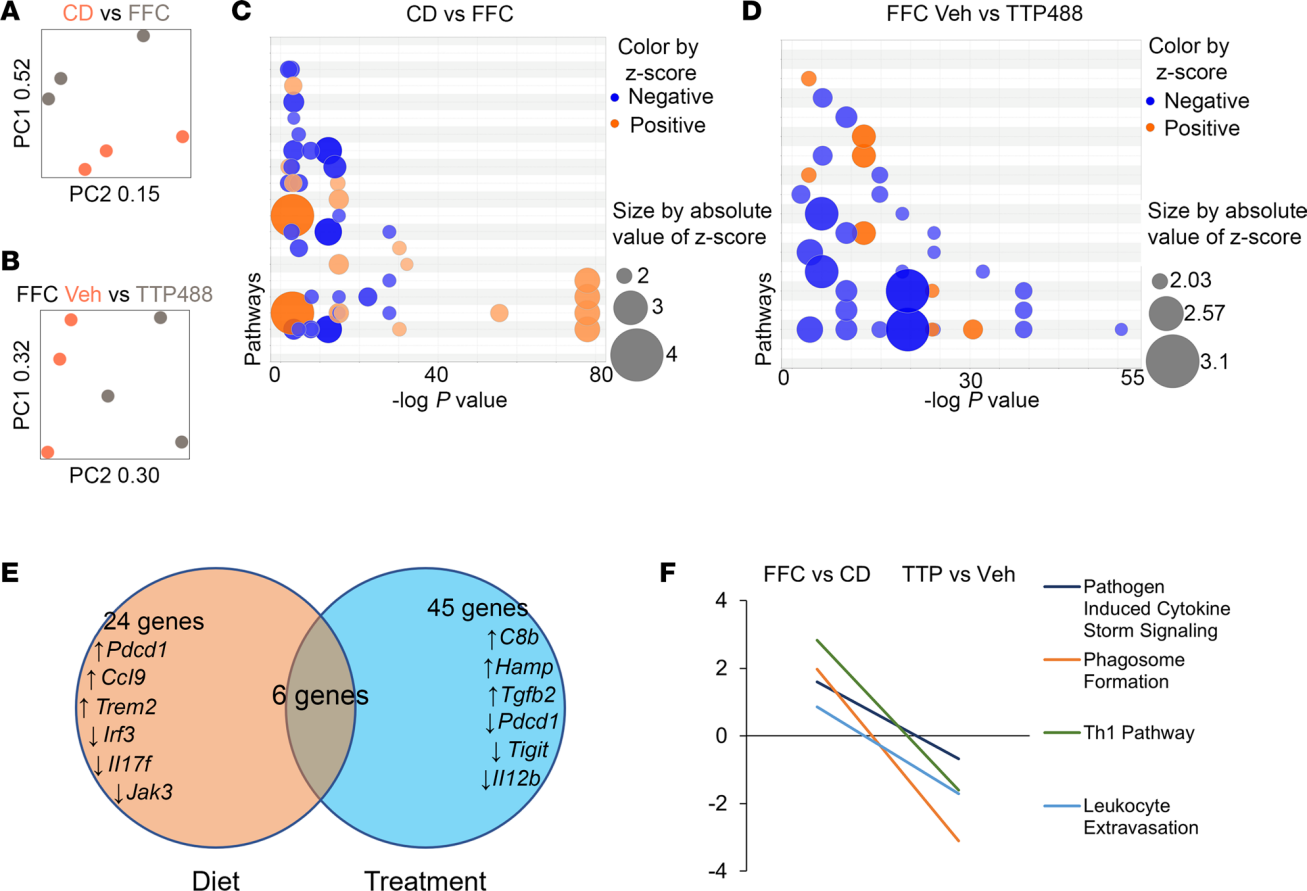
TTP488 attenuates diet-induced changes in the intrahepatic immune transcriptome. (**A**) Principal component analysis plots comparing transcriptome of IHLs from chow and FFC mice (*n* = 3 each). (**B**) Vehicle- and TTP488-treated FFC mice (*n* = 3 each). (**C**) Bubble plot of IPA canonical pathways significantly upregulated (orange) and downregulated (blue) with FFC diet (*n* = 3 each). (**D**) TTP488 treatment (*n* = 3 each). (**E**) Venn diagram depicting overlap of significantly different mRNA transcripts common to the experimental comparisons of diet and treatment. (**F**) Representative IPA canonical pathways that were significantly differentially regulated by FFC diet and TTP488 treatment.

To determine the functional state of IHLs in greater detail, we subjected RNA extracted from all isolated IHLs from FFC and chow mice to a NanoString array that included 581 immune-related transcripts. Principal component analysis identified overall separation of mRNA expression patterns based on diet (Figure 5A) as well as treatment (Figure 5B). Based on a FDR adjusted *P* value cutoff of < 0.05, 24 differentially expressed transcripts were identified in FFC mice compared with chow mice, of which 19 were upregulated and 5 were downregulated (Supplemental Table 2). Comparison of TTP488-treated FFC mice with vehicle-treated FFC mice demonstrated differential expression of 45 transcripts, of which 35 were upregulated and 10 were downregulated (Supplemental Table 2). Ingenuity pathway analysis (IPA) was utilized to understand the functional effects of these differentially expressed genes. Based on canonical pathway analysis, 11 pathways were significantly activated and 14 were significantly inhibited in FFC compared with chow mice (Figure 5C and Supplemental Table 3). Conversely, 4 pathways were significantly activated and 12 were significantly inhibited in TTP488- compared with vehicle-treated mice (Figure 5D and Supplemental Table 3); 4 of these pathways overlapped with diet-based differences. Based on these pathways, genes that were inversely influenced by diet and drug treatment were examined, revealing macrophage and T cell function–related transcripts (Figure 5E). Indeed, with stringent cutoffs to identify strength and significance of association, the pathways influenced by these genes (Pathogen Induced Cytokine Storm Signaling, Phagosome Formation, Th1 Pathway, Leukocyte Extravasation) that were activated by FFC diet and inhibited by TTP488 treatment were identified (Figure 5F). Taken together, flow cytometry and gene expression data of IHLs demonstrate an increase in RAGE^+^ recruited macrophages and activation of macrophage- and T cell–mediated proinflammatory pathways in FFC mouse livers. Conversely, there was a reduction in RAGE^+^ recruited macrophages and proinflammatory macrophage and T cell pathways with TTP488 treatment. Due to the lack of cell specificity with TTP488, we examined macrophages and T cells further by mass cytometry.

### Mass cytometry identifies subsets of RAGE^+^ macrophages influenced by TTP488.

**Figure 6:**
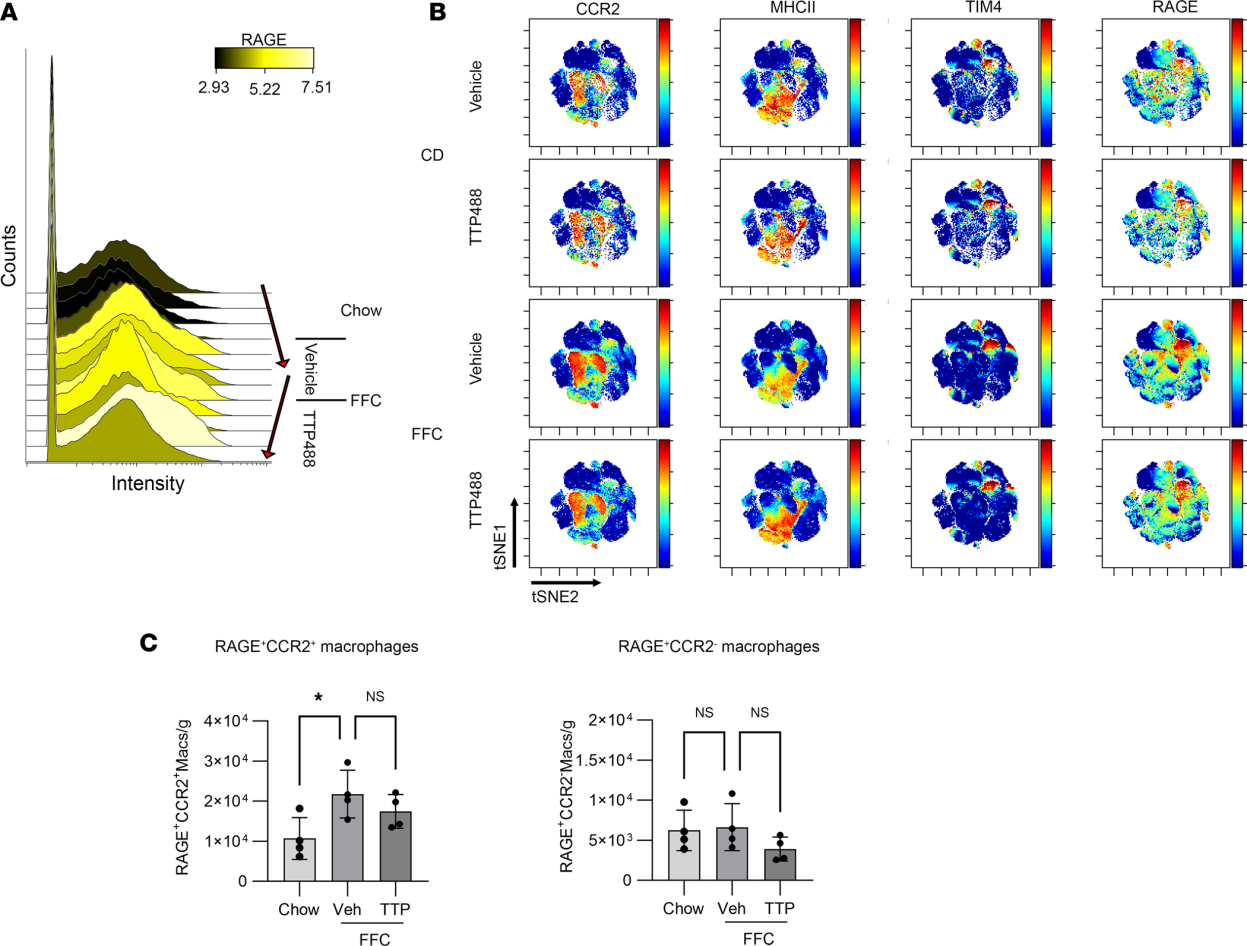
Mass cytometry identifies subsets of RAGE-expressing macrophage influenced by TTP488. (**A**) Histograms depicting changes in RAGE expression on macrophages (CD45^+^F4/80^+^) with FFC diet and TTP488 treatment (*n* = 4 each). (**B**) viSNE plots depicting expression of representative surface-markers. (**C**) Quantification of RAGE^+^CCR2^+^ (*n* = 4 each; *P* < 0.05) and RAGE^+^CCR2^–^ macrophages (*n* = 4 each; *P* = 0.7). Cell numbers are presented as cell count per gram of liver. Mann-Whitney *U* test was used for statistical analyses, and *P* values were adjusted by the Benjamini-Hochberg method for multiple comparisons.

**Figure 7:**
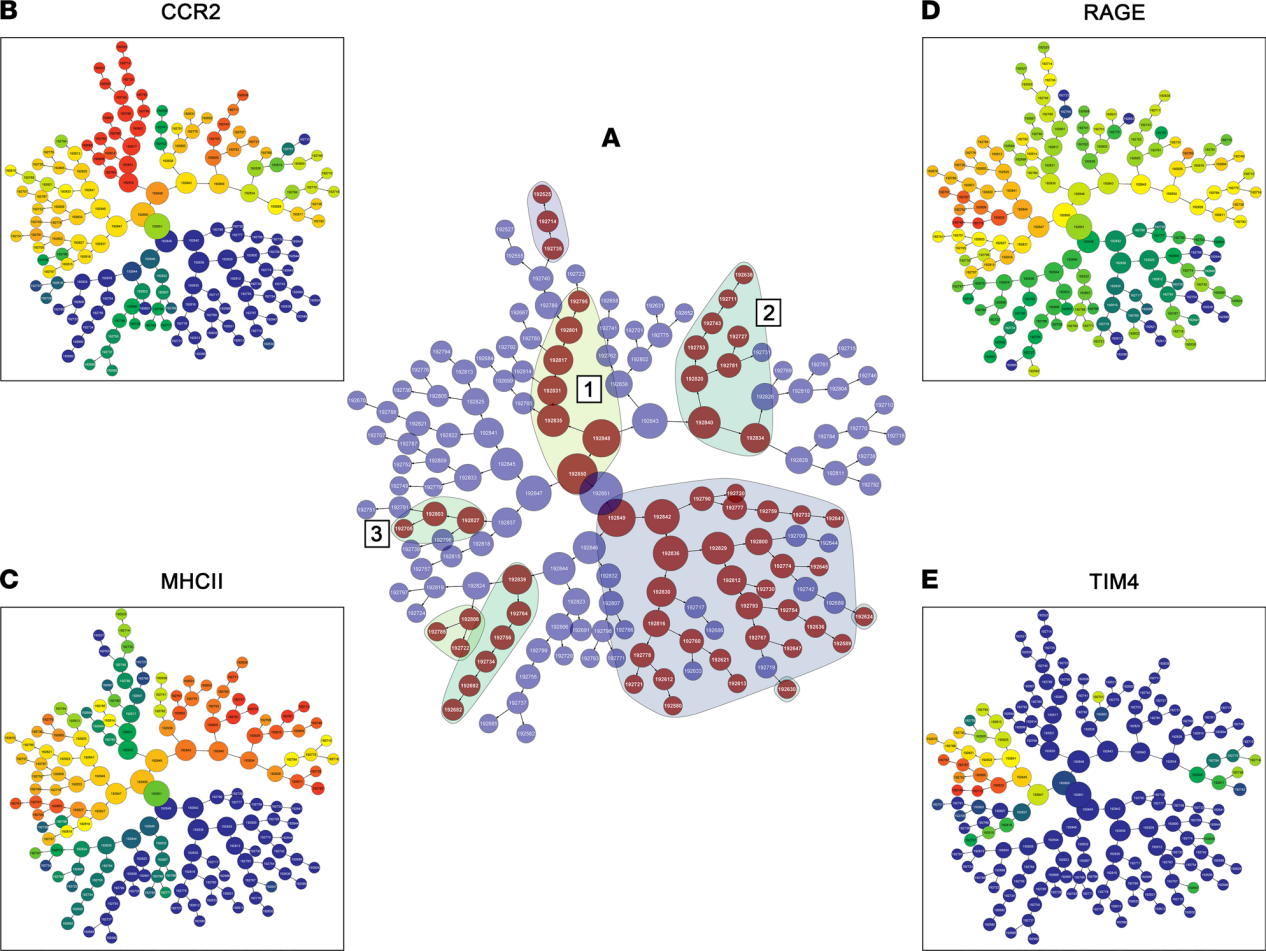
Unbiased hierarchical clustering of macrophages reveals distinct RAGE-enriched subsets. (**A**) CITRUS hierarchical tree with shaded groups of clusters that are differentially abundant between the experimental groups. The numbered groups (denoted as 1–3) represent clusters with RAGE enrichment. (**B**–**E**) Differential expression of representative surface markers colored based on intensity of expression are depicted in CCR2 (**B**), MHCII (**C**), RAGE (**D**), and TIM4 (**E**).

To investigate changes in IHL subsets and the macrophage–T cell relationship in greater depth, we performed mass cytometry or CyTOF on IHLs from chow and FFC mice following TTP488 and vehicle treatment. Following manual gating to identify CD45^+^CD3^–^CD19^–^F4/80^+^ cells as macrophages (Supplemental Figure 4), intensity and abundance of RAGE^+^ macrophages were both increased in FFC mice and reduced in TTP488-treated FFC mice (Figure 6A). In contrast to flow cytometry, multiple macrophage markers can be examined simultaneously using CyTOF. Therefore, in the analysis, rather than limiting to relative expression of F4/80 and CD11b to differentiate between recruited and resident macrophages, we visualized RAGE expression along with established markers of macrophage subsets, such as T cell immunoglobulin and mucin domain containing 4 (TIM4^+^), which is expressed on resident macrophages, and C-C motif chemokine receptor 2^+^ (CCR2^+^), which is expressed on recruited macrophages. On the viSNE plots, RAGE expression was present on both recruited (CCR2^+^) as well as resident (TIM4^+^) macrophages (Figure 6B), though it was predominantly detected in the CCR2^+^ populations. Indeed, concordant with earlier results from flow cytometry, RAGE^+^CCR2^+^ (a subset of recruited) but not RAGE^+^CCR2^–^ (a subset of resident) macrophages were increased in FFC mice, and both were numerically reduced with TTP488 treatment (Figure 6C). Furthermore, the abundance of RAGE^+^CCR2^+^ macrophages was greater than RAGE^+^CCR2^–^ macrophages, with a median abundance of 2 × 10^4^ versus 5 × 10^3^ cells/g of liver, respectively. To further define the expression of RAGE on recruited and resident macrophages, we performed unbiased hierarchical clustering of all CD45^+^CD3^–^CD19^–^F4/80^+^cells, encompassing all macrophages with CITRUS (cluster identification, characterization, and regression) and employing several known macrophage markers. First, all F4/80^+^ cells were clustered based on the expression of 14 macrophage markers (CD11b, CCR2, Ly6C, TIM4, TREM2, CD206, CX3CR1, MHCII, Ki67, NK1.1, RAGE, PD1, IRF5, IRF7) to identify clusters that were differentially abundant between the chow, FFC vehicle-, and FFC TTP488-treated mice (Figure 7A). Next, expression levels of representative macrophage markers (CCR2, MHCII, and TIM4) as well as RAGE on these clusters were visualized to identify RAGE^+^ macrophages that were also differentially abundant (Figure 7, B–E). This identified that 3 subsets of RAGE enriched macrophages were increased in FFC mice, denoted 1–3 (Figure 7A). These subsets were then examined further to understand their marker expression (Supplemental Figure 5, A–C). The first subset was CCR2^hi^, Ly6C^hi^, and CX3CR1^hi^ — potentially representing early recruited monocytes. The second subset was CCR2^hi^ but Ly6C^lo^, possibly established recruited macrophages, and the third was Ly6C^lo^, TIM4^hi^, and TREM2^hi^, resembling monocyte-derived resident macrophages, including the macrophage populations recently described by others and called NASH- and lipid-associated macrophages (19, 20). Interestingly, these 3 subsets appeared to differ in their proliferation rates, as suggested by the progressive decline in Ki67^+^ from subset 1 to 3 (Supplemental Figure 5D), consistent with their potential differentiation into more resident-like cells. Thus, RAGE expressing recruited macrophages across the different described stages of their occupation of the liver macrophage niche are increased in FFC diet. Next, we sought to examine the functional state of these RAGE^+^ macrophage subsets.

### RAGE activation on macrophages leads to induction of IRFs.

**Figure 8:**
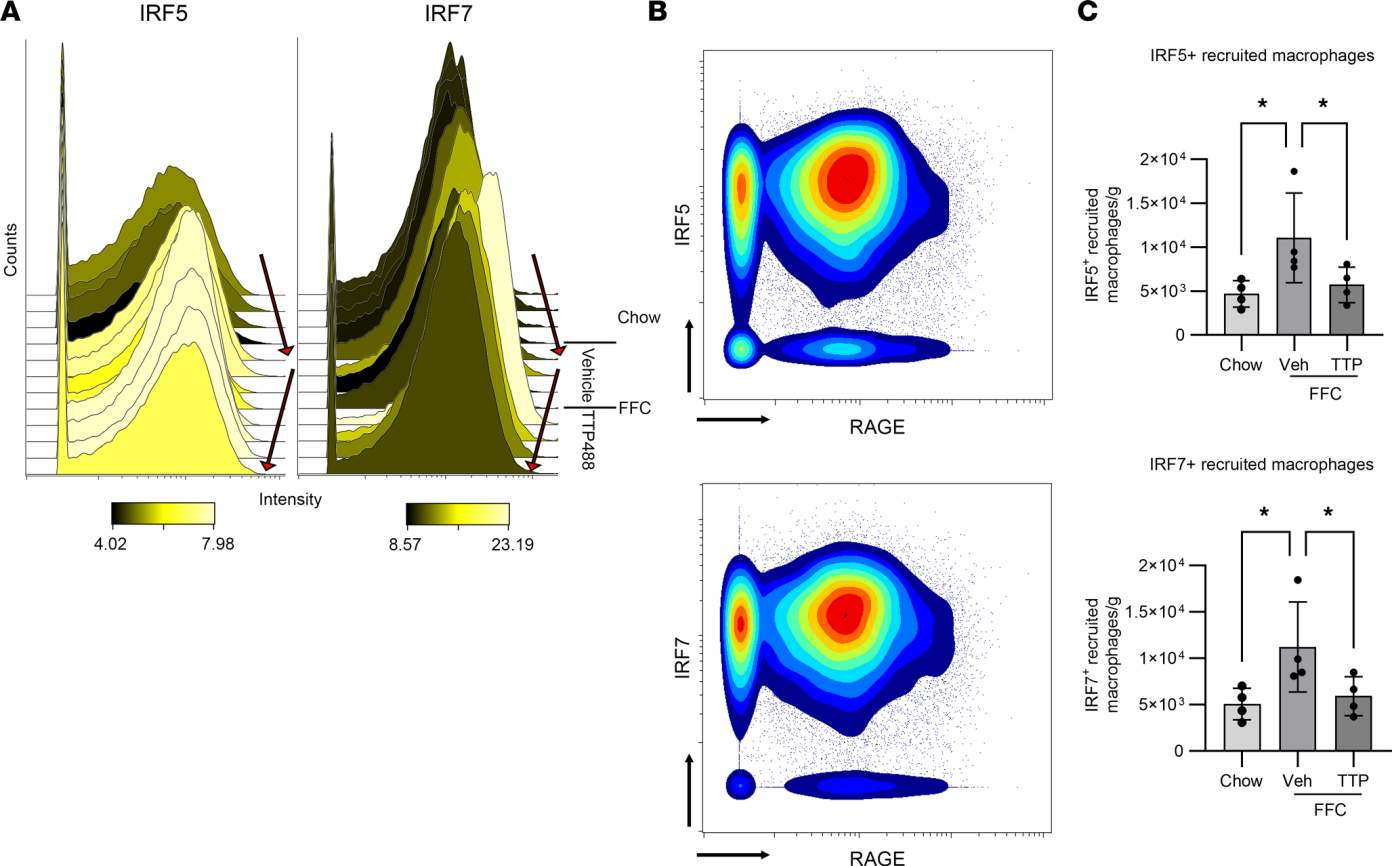
RAGE activation on macrophages is associated with induction of IRFs. (**A**) Histograms depicting expression of IRF5 and IRF7 on macrophages (CD45^+^F4/80^+^) assessed by CyTOF with FFC diet and TTP488 treatment. The arrows denote an increase in RAGE count and intensity among FFC vehicle compared with chow and a decrease in FFC TTP488. (**B**) Representative CyTOF biplots depicting coexpression of RAGE and IRF5 and IRF7 among recruited macrophages (CD45^+^/F4/80^+^/MHCII^+^TIM4^–^ cells); (**C**) Quantification of abundance of IRF5^+^ and IRF7^+^ recruited macrophages (CD45^+^/F4/80^+^/MHCII^+^TIM4^–^ cells) (*n* = 4 each; *P* < 0.05). Cell numbers are presented as cell count per gram of liver. Mann-Whitney *U* test was used for statistical analyses, and *P* values were adjusted by the Benjamini-Hochberg method for multiple comparisons.

To identify pathways affected by inhibition of RAGE that are known to be relevant for macrophage function, we examined the list of differentially expressed transcripts from the NanoString results. IFN regulatory factors (IRFs) are crucial mediators of the innate immune response and have a known pathogenic role downstream of RAGE and in NAFLD (21–23). Consistent with changes in mRNA expression, at the protein level, both intensity and abundance of IRF5^+^ and IRF7^+^ macrophages were increased in FFC mice and decreased with TTP488 treatment by CyTOF (Figure 8A). Next, we sought to identify which macrophage subset was driving these changes in IRF expression. Indeed, among recruited macrophages (CD45^+^/F4/80^+^/MHCII^+^TIM4^–^ cells), IRF5 and IRF7 were coexpressed in the RAGE^+^ population (Figure 8B). The abundance of IRF5^+^ and IRF7^+^ recruited macrophages was increased in FFC mice and significantly decreased with TTP488 treatment (Figure 8C). Thus, in FFC mice, induction of these proinflammatory transcription factors occurred in recruited macrophages concomitantly with RAGE activation and is reduced following RAGE inhibition, supporting a proinflammatory function for RAGE^+^ recruited macrophages. Given we had observed attenuated T cell activation with RAGE inhibition, we wanted to clarify whether this was mediated via macrophage RAGE signaling or directly by RAGE expression on T cells.

### Changes in T cell subsets are not RAGE dependent.

**Figure 9:**
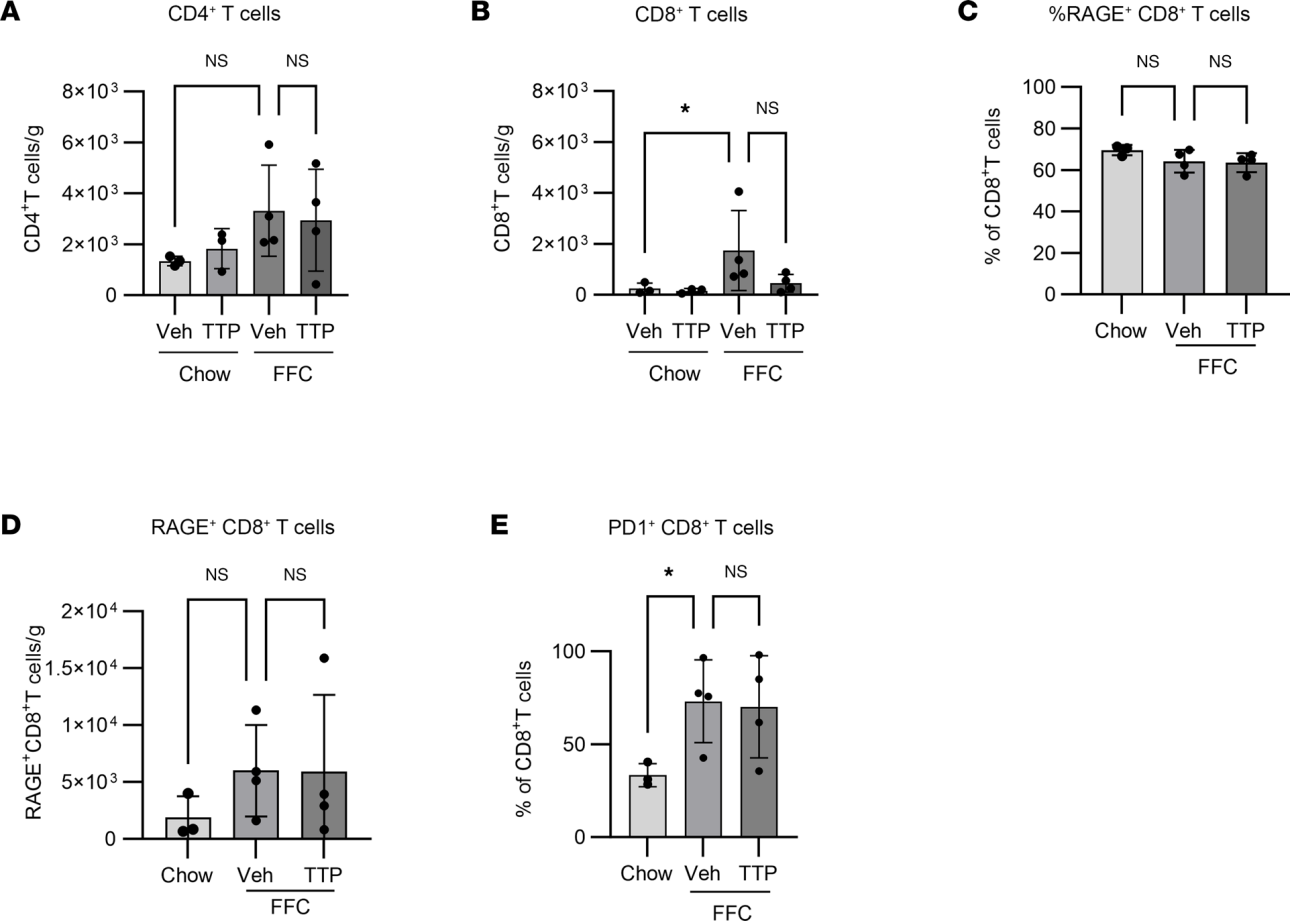
RAGE-expressing T cell subsets are unchanged by RAGE inhibition. (**A** and **B**) Flow cytometry based quantification of CD4^+^ T cells (Chow *n* = 3 each, FFC *n* = 4 each; *P* = 0.07) (**A**) and CD8^+^ T cells (Chow *n* = 3 each, FFC *n* = 4 each; *P* < 0.05) (**B**). (**C**–**E**) CyTOF-based quantification of percentage of RAGE^+^ among CD8^+^ T cells (Chow *n* = 3 each, FFC *n* = 4 each; *P* = 0.2) (**C**); RAGE^+^CD8^+^ T cells (Chow *n* = 3 each, FFC *n* = 4 each; *P* = 0.4) (**D**); and PD1^+^CD8^+^ T cells (Chow *n* = 3 each, FFC *n* = 4 each; *P* < 0.05) (**E**). Cell numbers are presented as cell count per gram of liver. Mann-Whitney *U* test was used for statistical analyses, and *P* values were adjusted by the Benjamini-Hochberg method for multiple comparisons.

Given the differentially expressed transcripts related to T cell function on the NanoString array, we examined T cells by flow cytometry and CyTOF. By flow cytometry, as expected, CD8^+^ but not CD4^+^ T cells were significantly more abundant in FFC mice compared with chow mice and were numerically reduced in TTP488-treated FFC mice (Figure 9, A and B). By CyTOF, although approximately 60%–70% of CD8^+^ cells expressed RAGE (Figure 9C), the absolute abundance of RAGE-expressing CD8^+^ T cells was unchanged with both diet and treatment, demonstrating that TTP488 did not reduce the number of RAGE^+^CD8^+^ T cells (Figure 9D). Concordant with the NanoString results, where *Pdcd1* was the top upregulated gene in FFC mice, the proportion of CD8^+^ cells that were PD1^+^ was significantly increased in FFC mice (Figure 9E). However, by viSNE analysis, these PD1^+^CD8^+^ cells included subsets expressing markers of T cell function such as BATF, TCF1, EOMES, and GranzB (Supplemental Figure 6). Thus, interpreting the changes in macrophage subsets along with the CD8^+^ T cell data, we hypothesized that changes in CD8^+^ T cells were driven indirectly, by crosstalk with RAGE^+^ macrophages. Indeed, upregulation of inflammatory IRFs downstream of RAGE activation on macrophages could mediate crosstalk with T cell subsets via release of type I IFN.

### Myeloid-specific RAGE KO attenuates diet-induced NASH.

Given the lack of cell type specificity with the use of TTP488 for RAGE inhibition, we utilized a genetic approach to delete RAGE specifically on BM myeloid cell precursors, which give rise to recruited macrophages. The genotype of RAGE-floxed mice crossed with LyZM Cre mice was confirmed by PCR (Supplemental Figure 7A). Next, the deletion of RAGE was confirmed by quantitative PCR (qPCR) and flow cytometry. *Ager* mRNA expression in BM-derived macrophages (BMDM) from RAGE–myeloid KO (RAGE-MKO) and littermate WT control mice demonstrated a significant reduction in RAGE expression in RAGE-MKO BMDM (Supplemental Figure 7B). Flow cytometry for EGFP, which is expressed following Cre-mediated recombination, demonstrated that EGFP was undetectable in WT and expressed in a majority of BMDM from RAGE-MKO mice (Supplemental Figure 7, C and D). Approximately 60% of all myeloid cells from the BM of RAGE-MKO expressed EGFP, as not all CD11b^+^ cells express *LyzM*, which is specific for myelomonocytic cells — monocytes, macrophages, and granulocytes in mice (24).

**Figure 10:**
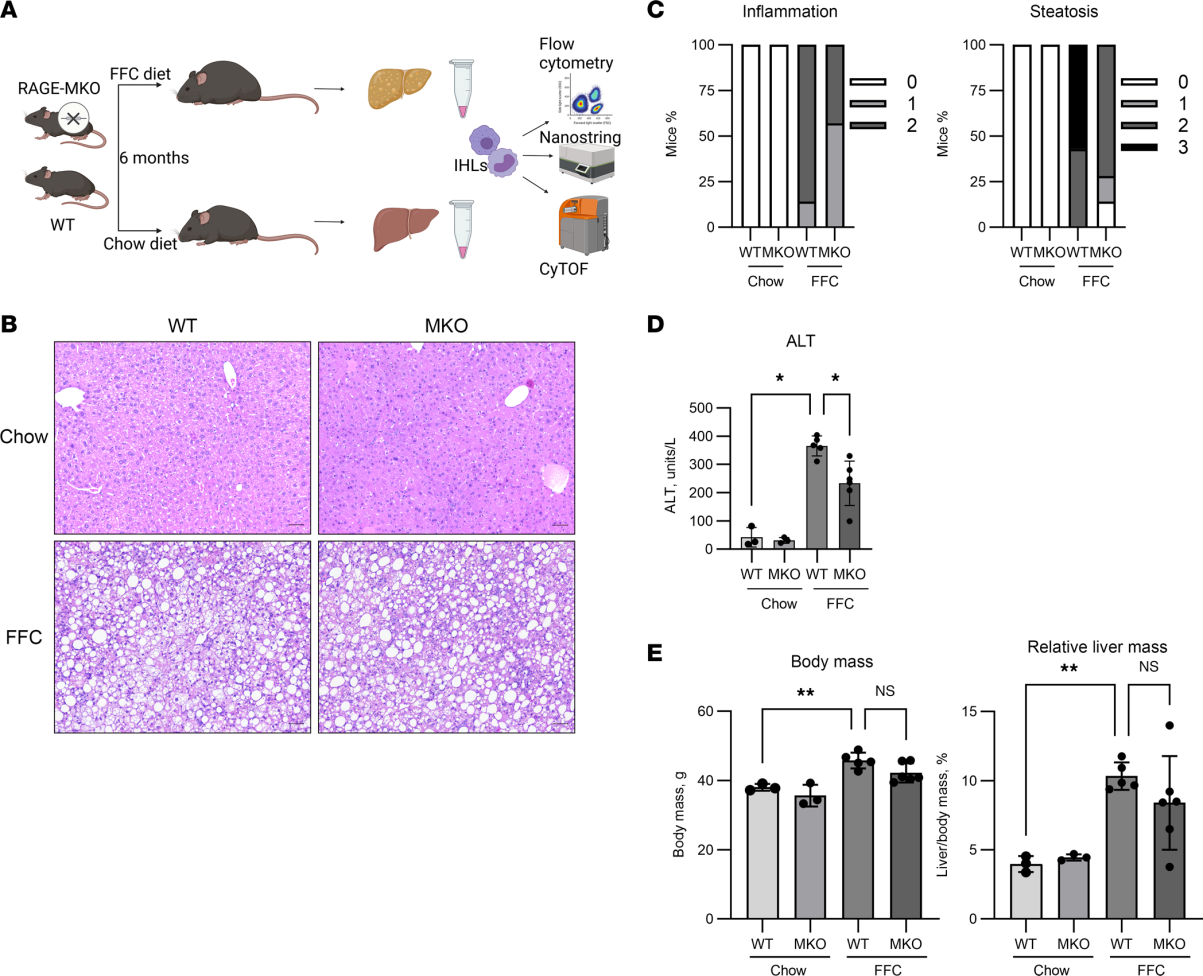
RAGE MKO attenuates NASH. (**A**) Schematic depicting experimental design for RAGE MKO. (**B**) Representative images of H&E-stained livers from the chow and FFC WT and MKO mice (Chow-WT *n* = 3, Chow-MKO *n* = 3, FFC-WT *n* = 5, FFC-MKO *n* = 6). Scale bar: 50 μm. (**C**) Distribution of inflammation and steatosis subscores of the NAFLD activity score. (**D**) Serum ALT (Chow-WT *n* = 3, Chow-MKO *n* = 3, FFC-WT *n* = 5, FFC-MKO *n* = 6; *P* < 0.05). (**E**) Body mass (Chow-WT *n* = 3, Chow-MKO *n* = 3, FFC-WT *n* = 5, FFC-MKO *n* = 6; *P* < 0.01). (**F**) Relative liver mass (Chow-WT *n* = 3, Chow-MKO *n* = 3, FFC-WT *n* = 5, FFC-MKO *n* = 6; *P* < 0.01). Mann-Whitney *U* test was used for statistical analyses, and *P* values were adjusted by the Benjamini-Hochberg method for multiple comparisons.

**Figure 11:**
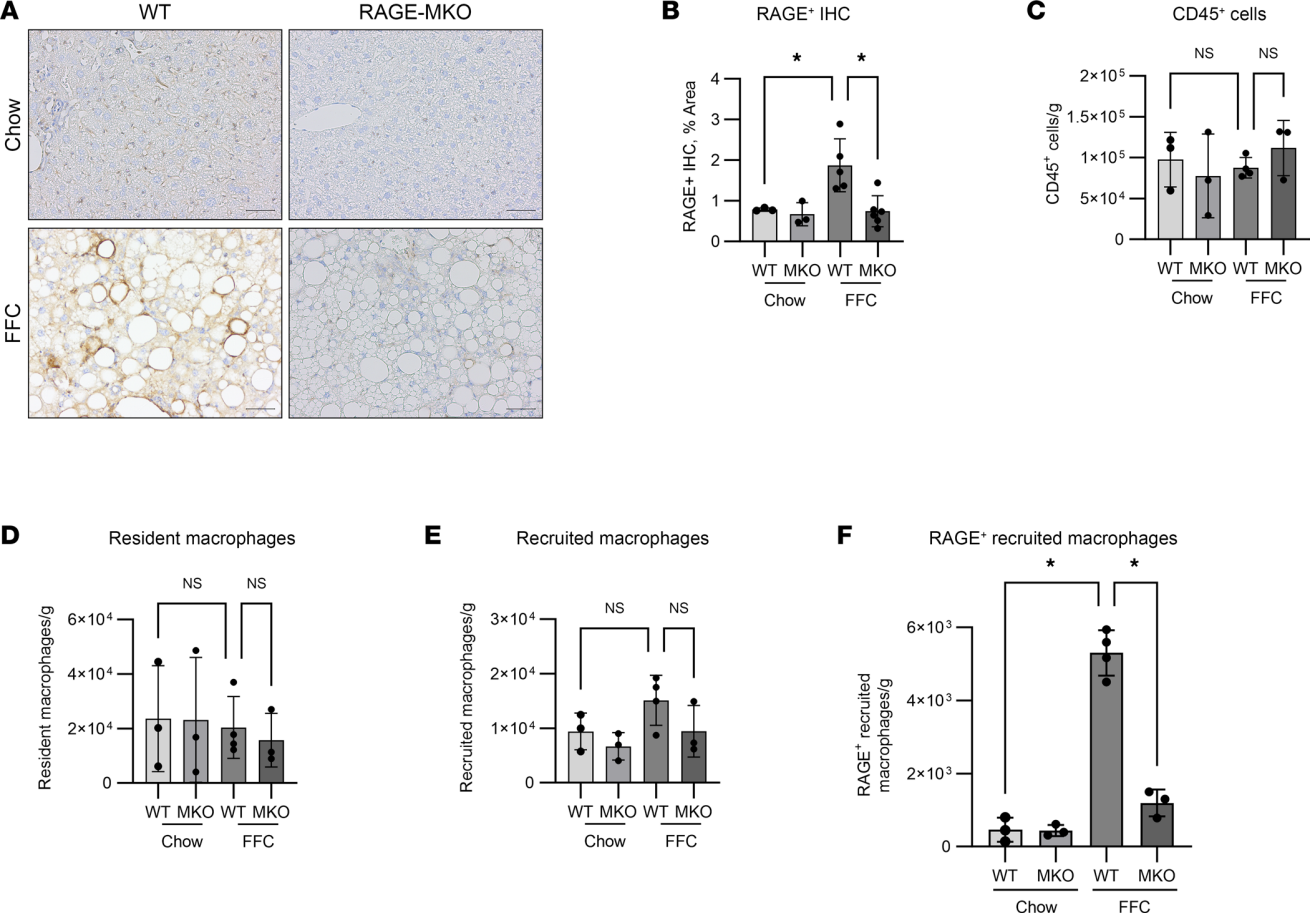
RAGE MKO reduces RAGE expression. (**A**) Representative images of RAGE IHC from chow and FFC WT and MKO mice. Scale bar: 50 μm. (**B**) Quantification of **A** (Chow-WT *n* = 3, Chow-MKO *n* = 3, FFC-WT *n* = 5, FFC-MKO *n* = 6; *P* < 0.05). (**C**) Quantification of CD45^+^ cells by flow cytometry (Chow-WT *n* = 3, Chow-MKO *n* = 3, FFC-WT *n* = 4, FFC-MKO *n* = 3; *P* = 0.7). (**D**) Quantification of resident macrophages (Chow-WT *n* = 3, Chow-MKO *n* = 3, FFC-WT *n* = 4, FFC-MKO *n* = 3; *P* = 0.9). (**E**) Quantification of recruited macrophages (Chow-WT *n* = 3, Chow-MKO *n* = 3, FFC-WT *n* = 4, FFC-MKO *n* = 3, *P* = 0.2). (**F**) Quantification of RAGE^+^ recruited macrophages (Chow-WT *n* = 3, Chow-MKO *n* = 3, FFC-WT *n* = 4, FFC-MKO *n* = 3; *P* < 0.05). Cell numbers are presented as cell count per gram of liver. Mann-Whitney *U* test was used for statistical analyses, and *P* values were adjusted by the Benjamini-Hochberg method for multiple comparisons.

Male RAGE-MKO and WT mice were fed either chow or FFC diet for 6 months (Figure 10A). The upregulation in RAGE expression in FFC mice in macrophages was confirmed by co-IF (Supplemental Figure 8, A–C), and predictably, it was not detected in RAGE-MKO mice (Supplemental Figure 8A). Compared with WT, FFC RAGE-MKO mice had attenuation of liver injury as assessed by H&E (Figure 10B), histological scoring of inflammation and steatosis according to the NAS (Figure 10C) and plasma ALT (Figure 10D). FFC WT and RAGE-MKO mice had similar body mass and relative liver mass (Figure 10E). The degree of fibrosis quantified by sirius red staining was significantly attenuated in FFC RAGE-MKO compared with WT mice (Supplemental Figure 9, A and B). By IHC, RAGE expression was significantly reduced in FFC RAGE-MKO compared with WT mice (Figure 11, A and B). By flow cytometry, the total numbers of CD45^+^ cells (Figure 11C), resident (CD11b^int^F4/80^hi^) (Figure 11D), and recruited (CD11b^hi^F4/80^int^) (Figure 11E) macrophages were similar in FFC RAGE-MKO and WT mice. However, compared with WT, RAGE^+^ recruited macrophages (Figure 11F) were reduced in FFC RAGE-MKO mice. Thus, RAGE-MKO mice, predictably, accumulated fewer RAGE^+^ macrophages in FFC mouse livers, while the total number of recruited macrophages was unchanged, suggesting an increase in the recruitment of other macrophage subsets. This correlation between reduced RAGE^+^ macrophage accumulation and reduction in liver injury and inflammation support a proinflammatory role for RAGE^+^ recruited macrophages in NASH.

### Myeloid-specific RAGE-KO attenuates macrophage and T cell inflammatory phenotype.

With the ability to assess downstream effects of macrophage-specific RAGE deletion in the RAGE-MKO model, we profiled the IHL transcriptome from RAGE-MKO mice with a NanoString array. Based on a FDR adjusted *P* value cutoff of < 0.01, a total of 62 differentially expressed transcripts were identified in FFC mice compared with chow mice, of which 21 were upregulated and 41 were downregulated (Supplemental Table 2). Canonical pathway analysis using IPA demonstrated that the top 5 pathways downregulated in RAGE-MKO compared with WT mice were Pathogen Induced Cytokine Storm Signaling Pathway, Th1 Pathway, IL-12 Signaling and Production in Macrophages, Th2 Pathway, and Macrophage Classical Activation Signaling Pathway (Supplemental Figure 10, A and B, and Supplemental Table 3). Hence, similar to pharmacological inhibition of RAGE, myeloid-specific RAGE deletion inhibited macrophage activation and T cell activation pathways. In-depth analysis of macrophage and T cell subsets by CyTOF, similar to the TTP488 data set, demonstrated that the abundance of RAGE-expressing recruited but not resident macrophages was decreased in FFC RAGE-MKO compared with WT mice (Supplemental Figure 11, A and B). Concordant with the absence of RAGE-expressing recruited macrophages and subsequent RAGE-activated downstream signaling, the numbers of IRF5 and IRF7 expressing recruited macrophages were also lower in RAGE-MKO compared with WT mice (Supplemental Figure 11, C and D). Next, we performed CITRUS-based clustering of all F4/80^+^ cells based on the expression of macrophage markers to identify clusters that were differentially abundant between the FFC RAGE-MKO and WT mice (Supplemental Figure 11E). Among clusters that were less abundant in RAGE-MKO mice (shaded area), the expression levels of representative macrophage markers were visualized, to confirm that RAGE^+^ recruited (CCR2^+^Ly6C^+^) macrophages were decreased in RAGE-MKO compared with WT mice (Supplemental Figure 11E). Examining T cell subsets, the abundance of CD8^+^ T cells was reduced in RAGE-MKO compared with WT mice (Supplemental Figure 11F). In contrast to the effect of FFC diet on CD8^+^ T cell subsets, in RAGE-MKO compared with WT mice, there was a reduced abundance of activated CD8^+^ T cell subsets expressing PD1^+^, GrzB^+^, and the recently described autoaggressive CXCR6^+^ (Supplemental Figure 11, G–I). Thus, macrophage RAGE deletion attenuates proinflammatory CD8^+^ T cell subsets.

### Myeloid-specific RAGE-KO attenuates macrophage-mediated CD8^+^ T cell activation in vitro.

**Figure 12:**
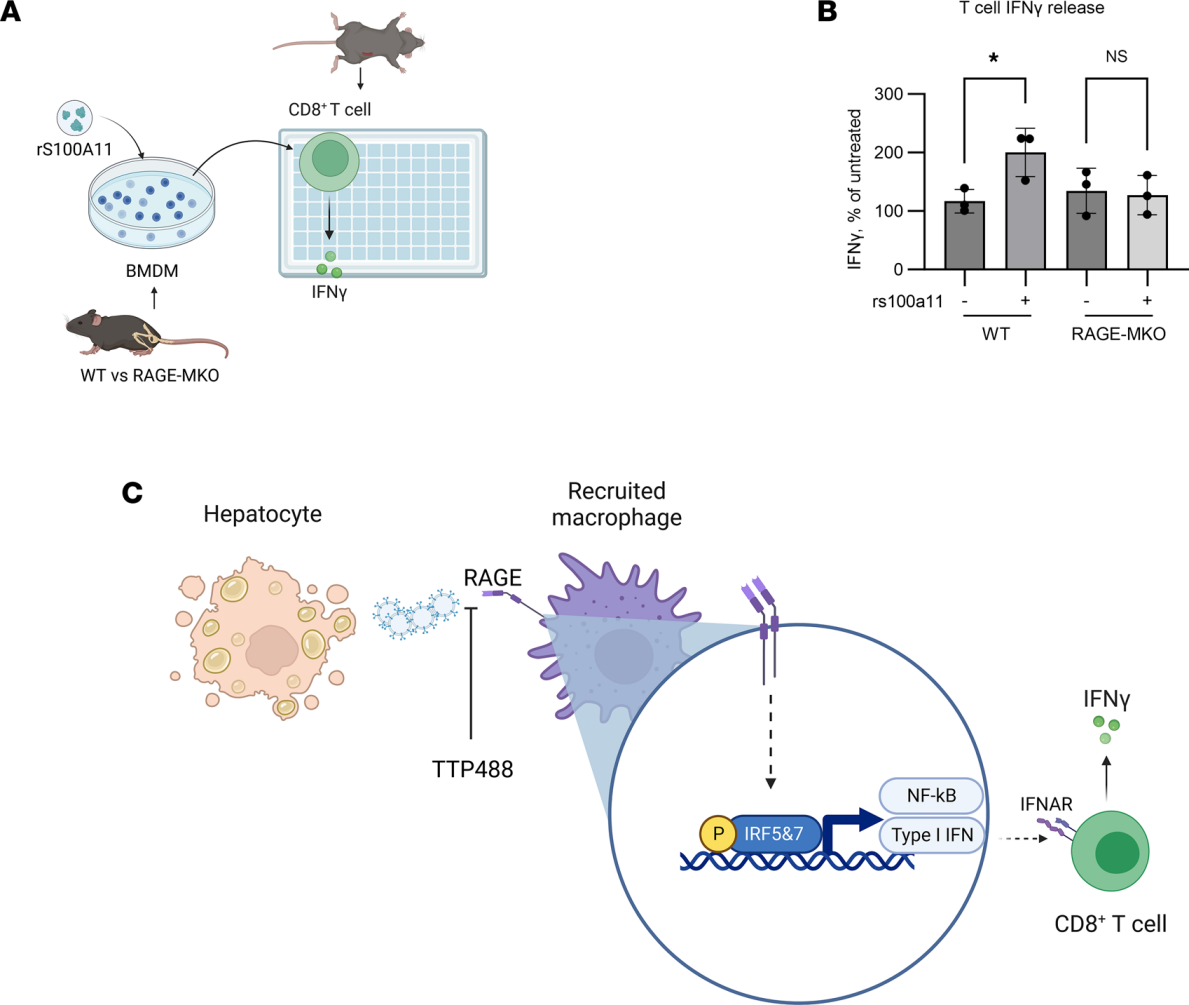
Macrophage RAGE signaling mediates proinflammatory crosstalk with CD8^+^ T cells. (**A**) Schematic depicting experimental design for demonstrating in vitro crosstalk between macrophages and CD8^+^ T cells. BM-derived macrophages (BMDM) isolated from WT or MKO mice were stimulated ex vivo with recombinant S100A11, a known RAGE agonist and the supernatant used to stimulate CD8^+^ T cells. Release of IFN-γ was measured by ELISA. (**B**) Quantification of IFN-γ release from CD8^+^ T cells measured by ELISA, normalized to untreated CD8^+^ T cells. The horizontal axis shows source of supernatant from treated BMDMs (*n* = 3 each; *P* < 0.05). Mann-Whitney *U* test was used for statistical analyses. (**C**) Proposed model depicting the proinflammatory role of RAGE^+^ macrophage–T cell crosstalk in NASH. Herein, we propose that RAGE upregulation occurs in human and murine NASH, specifically on recruited macrophages. RAGE activation on recruited macrophages, potentially by DAMPs, leads to upregulation of IRFs, which may mediate proinflammatory crosstalk with T cells. Interruption of RAGE with a pharmacological inhibitor or with RAGE MKO is sufficient to ameliorate NASH.

In order to confirm RAGE-dependent macrophage–CD8^+^ T cell crosstalk, we stimulated BMDM from male WT and RAGE-MKO mice with recombinant S100A11 (rS100A11), a known RAGE ligand (25, 26) (Figure 12A). Supernatant from these treated macrophages was used to stimulate CD8^+^ T cells from a WT mouse. Compared with WT macrophages, supernatants from RAGE-MKO macrophages had an attenuated capacity to induce the release of IFN-γ from CD8^+^ T cells (Figure 12B), thus supporting the model that RAGE activation on macrophages mediates inflammatory crosstalk with CD8^+^ T cells (Figure 12C).

## Discussion

Liver inflammation in NASH is characterized by the activation of macrophages that are expanded due to the recruitment of proinflammatory monocyte-derived macrophages (1–3). Herein, we demonstrate the role of RAGE-expressing recruited macrophages as a mediator of the inflammatory intrahepatic milieu in NASH. Firstly, we show that RAGE is upregulated in human as well as mouse models of NASH, specifically on recruited macrophages in the liver. Secondly, a pharmacological inhibitor of RAGE, TTP488 reduced the intrahepatic accumulation of RAGE-expressing macrophages and ameliorated liver injury. Thirdly, transcriptomics analysis of IHLs suggested that RAGE inhibition attenuated FFC diet–induced activation of proinflammatory macrophage and T cell pathways. By mass cytometry, we identified that RAGE expression on distinct subsets of recruited macrophages was accompanied by activation of IRF5 and IRF7 that could mediate crosstalk with T cells. In keeping with this, the myeloid cell–specific deletion of RAGE was sufficient to attenuate liver injury and inflammation in FFC mice and attenuate the activation of CD8^+^ T cells in vitro. Altogether, our data suggest an upstream role of RAGE on recruited macrophages as a mediator of proinflammatory immune activation contributing to liver injury in NASH.

RAGE is a pattern-recognition receptor that is known to be induced in macrophages in obesity and atherosclerosis (8, 9) and is a putative receptor for several DAMPs released as a consequence of lipotoxic hepatocyte injury (8, 26), including S100A11, which we have demonstrated to be enriched on lipotoxic extracellular vesicles (27). We discovered that hepatic macrophage RAGE expression is significantly upregulated in human and murine NASH. While RAGE can be expressed on many cell types, employing complementary techniques of co-IF, flow cytometry, and mass cytometry, we demonstrate that FFC diet–induced induction of RAGE expression occurred on recruited macrophages. Recently, the importance of hepatocellular RAGE signaling in response to a diet high in AGEs was demonstrated (13). Our data from myeloid cell–specific RAGE deletion and pharmacological inhibition are complementary to these observations and suggest a phasic or simultaneous activation of RAGE — on macrophages potentially by DAMPs released due to lipotoxicity and on hepatocytes by AGEs produced as a consequence of hyperglycemia.

RAGE is a promiscuous receptor with several ligands; therefore, we first took a ligand-independent approach by administering a pharmacological antagonist of the extracellular domain of RAGE (TTP488) to mice and subsequently confirmed the role of RAGE signaling in recruited macrophages in NASH in RAGE-MKO mice. TTP488 administration and RAGE-MKO led to amelioration of liver injury and reduced the accumulation of RAGE-expressing recruited macrophages. There was no effect on steatosis and other metabolic parameters in these mice, consistent with a previous report of global RAGE KO (28). The degree of fibrosis attenuation was not statistically significant in TTP488-treated mice, which could possibly be due to the duration of treatment, since fibrosis was significantly reduced in RAGE-MKO mice. Notably, TTP488 has a known acceptable safety profile for use in humans, has previously been studied in Phase III clinical trials for Alzheimer’s disease, and may have potential as a therapeutic agent in NASH (29). While the salutary effects of TTP488 could be due to inhibition of RAGE signaling on multiple cell types, the data demonstrating a reduction in liver injury, inflammation, and fibrosis in MKO mice compared with littermate floxed controls demonstrates a role for RAGE in BM-derived myeloid cells.

Recently, there have been several advances in the understanding of the heterogeneity of hepatic macrophages (30). Given macrophage-specific upregulation of RAGE in NASH, we investigated the role of RAGE in mediating macrophage identity and function. We used an unbiased hierarchical clustering method with 14 macrophage markers to identify RAGE-expressing subsets that were differentially abundant between FFC and chow mice. This revealed that RAGE expression persists across the trajectory of recruitment of monocytes (Ly6C^hi^) to the NASH liver, where these monocytes repopulate the resident macrophage niche (TIM4^hi^) (31). Furthermore, there appears to be a progressive loss in proliferative capacity with RAGE^+^ recruited macrophages expressing less Ki67 than RAGE^+^ monocytes, suggesting a constant replenishment by recruited monocytes (32).

Broader examination of the transcriptome of IHLs in chow and FFC mice administered vehicle or TTP488 identified an upregulation of macrophage genes known to be upregulated in NASH — for example, *Trem2* (20), *Ccr2* (33), and *Cx3cr1* (7) — in FFC mice and a reduction following TTP488 administration. Additionally, markers of T cell function, including *Tigit*, *Runx3*, and *Pdcd1*, which encodes PD-1 and was recently described to be upregulated on auto-aggressive T cells in NASH, were similarly noted to be inversely related, elevated in FFC, and lowered in TTP488-treated mice (34, 35). Canonical pathway analysis using IPA identified that macrophage- and T cell–mediated proinflammatory pathways were activated in FFC mouse livers and reduced with TTP488 treatment. Mirroring these findings, the top pathways downregulated in RAGE-MKO compared with WT mice were all related to macrophage and T cell activation. Thus, transcriptomics profiling of IHLs reveal that RAGE antagonism with TTP488 as well as myeloid RAGE deletion reverses macrophage and T cell activation pathways in diet-induced murine NASH. Combining these observations with the findings from MKO mice, we propose that macrophage RAGE activation leads to T cell responses via IRF5 and IRF7, which were both increased in RAGE-expressing macrophages in FFC mice and reduced following TTP488 administration. These findings are consistent with the known proinflammatory roles for IRF5 (36) and IRF7 (37) in obesity-associated inflammation and their activation downstream of RAGE signaling. Furthermore, the increase in CD8^+^ T cells with FFC diet was attenuated in TTP488- compared with vehicle-treated mice. By CyTOF, we could confirm that PD1^+^CD8^+^ T cells were indeed increased with FFC diet and included several effector and exhausted subsets (38). Although CD8^+^ T cells expressed RAGE, consistent with a described role of RAGE on T cells (39), RAGE-expressing T cells were unchanged with FFC diet and treatment, suggesting that the effect of RAGE activation and inhibition was mediated indirectly, potentially via RAGE-expressing recruited macrophages. Given that IRF can mediate secretion of soluble type I IFN, such as IFN-α, that can bind to the receptor IFNAR expressed on pathogenic T cells in NASH (40) and can induce PD-1 expression on T cells (41), we examined macrophage–T cell crosstalk in vitro; our data confirm that the secretome of WT and not RAGE-KO macrophages activated by S100A11 leads to downstream T cell activation.

In conclusion, we demonstrate that RAGE upregulation occurs in human and murine NASH, where it occurs on recruited macrophages. Interruption of RAGE signaling with a pharmacological inhibitor or myeloid-specific RAGE KO is sufficient to ameliorate NASH. Based on prior studies demonstrating the pathogenic role of RAGE and type I IFN signaling in NASH, we demonstrate that RAGE influences IRF signaling on recruited macrophages, which may mediate crosstalk with T cell subsets that further perpetuate liver injury. This highlights macrophage-specific RAGE signaling as a key node for pathogenic intercellular signaling with potential as a powerful therapeutic target in NASH.

## Methods

### Mouse studies.

Nine-week-old C57BL/6J male mice were purchased from The Jackson Laboratory and were acclimated for 3 weeks, during which all mice received standard rodent chow diet. Mice had unrestricted access to food and water and were housed in standard pathogen–free facilities with 12:12-hour day-night circadian cycles. Cohorts of mice were fed 2 different diets to induce hepatic steatosis and inflammation. Dietary NASH groups were as follows. (a) FFC diet: Adult male mice were assigned to 2 groups of similar starting weight, to receive chow or FFC diet, which provides 40% kcal from fat and is composed of 34% sucrose, 20% milk fat, and 0.21% cholesterol (AIN-76A Western Diet, originally manufactured as D12079B, TestDiet). Mice in the FFC group also received sweetened drinking water (23.1 g/L fructose and 18.9 g/L glucose) ad libitum. This murine model results in the development of insulin resistance, adipose tissue inflammation, and NASH with hepatocyte ballooning and fibrosis, displaying high fidelity to human NASH (15, 16). After 20 weeks on the diet, by which time liver inflammation is established (42), mice in each group were randomly split into treatment arms — either with drug (TTP488, Selleckchem), dissolved as per manufacturer recommendations in 10% DMSO, 40% PEG300, 5% Tween 80, and 45% ddH_2_0), or vehicle alone. Mice received daily i.p. injections of drug (4 mg/Kg), a dose reported to be efficacious for in vivo immune modulation of murine T cells (18), or equivalent volume of vehicle for 30 days, while they continued to be on their respective diets. (b) MCD diet: Adult male mice weighing 19–24 g were assigned to 2 groups of similar starting weight, to be fed either a MCD (catalog A02082002Bri) diet or its control MCS (catalog A02082003Byi, Research Diets). Although this model is characterized by the development of steatosis and liver inflammation in a relatively short period of time (approximately 4 weeks), the systemic metabolic profile, including loss of body weight and the preserved insulin sensitivity tolerance, do not faithfully mirror human NASH (14).

Upon completion of the study, mice were euthanized following a 6-hour fast. Platelet-poor plasma was isolated from blood collected by cardiac puncture by centrifugation at 2,000*g* for 20 minutes, followed by 13,000*g* for 2 minutes at 4°C. Plasma was stored at −80°C until further analyses. The liver was excised, weighed, and apportioned for downstream analyses, including RNA extraction, protein extraction, formalin fixation for histology, and intrahepatic leukocytes (IHL) isolation. IHLs were employed for RNA extraction, as well as flow cytometry and CyTOF.

### RAGE-MKO model.

*Rage^fl/fl^* mice have been previously described (28, 43) and were a gift from Natalie Nieto (Department of Pathology, University of Illinois at Chicago, Chicago, Illinois, USA). We crossed *Rage^fl/fl^* mice with LyzM-Cre transgenic mice (The Jackson Laboratory, strain no. 004781) in which the Cre recombinase is expressed under the control of an endogenous lysozyme 2 gene (*Lyz2*) promoter. This construct is designed such that Cre recombination results in RAGE deletion and simultaneous activation of a reporter gene (*Egfp*) transcription (43). Exons 2–7 of the *Ager* gene are flanked by 2 *loxP* sites in the same orientation, so upon exposure to Cre, the intervening genomic sequences are deleted. Subsequently, the thymidine kinase (TK) promoter is moved directly in front of the start site of the promoterless *Egfp* open reading frame. The LyzM-Cre approach has been used to specifically target myeloid subsets, as this leads to deletion of floxed genes in BMDM but not embryonic liver resident macrophages (44). RAGE myeloid-specific KO (*Rage^fl/fl^ LyzM^Cre/0^*), referred to as RAGE-MKO, and littermate WT controls (WT, *Rage^fl/fl;0/0^*) were fed either chow or FFC for 6 months to induce NASH. Both Cre expression and RAGE KO were confirmed in the RAGE-MKO compared with WT mice (Supplemental Figure 6A). After euthanasia, plasma and liver samples were collected as above, under mouse studies.

### Measurement of metabolic parameters.

Glucose tolerance tests were performed after a 12-hour fast as previously described (45). Briefly, glucose tolerance was measured after i.p. injection of 2 g glucose/kg body weight to overnight-fasted animals. Blood glucose was measured using a OneTouch Ultra glucometer (LifeScan Inc.) with a sensitivity of 10 mg/dL, at 0, 15, 30, 45, 60, 90, and 120 minutes after injection. An AUC for glucose concentration was calculated and compared between treatment groups. Metabolic parameters in individual FFC mice were measured as previously described (46); lean mass and fat mass were quantified using computed tomography (EchoMRI; LaTheta) and oxygen consumption (V̇o_2_) and carbon dioxide production (V̇co_2_) measured using the comprehensive laboratory animals monitoring system (CLAMS), equipped with an Oxymax Open Circuit Calorimeter System (Columbus Instruments). Resting, activity, and total energy expenditure; 24-hour food intake; heat production; and physical activity in the horizontal (ambulation) and vertical (rearing) planes were also measured for a 48-hour period in the CLAMS using photocells.

### Histological analyses.

Sections (5 μm) of formalin-fixed, paraffin-embedded (FFPE) liver tissues were stained with H&E using standard techniques and used for histological grading, according to the NAS. Fibrosis was assessed by sirius red staining in 5 μm liver sections, as described previously (47). IHC was performed following the standard ABC Immunostaining kit (Vector Laboratories) protocol and using primary antibodies for Mac-2 (1:250 dilution; 14–5301-82, eBiosciences) and RAGE (1:100 dilution; sc-365154, Santa Cruz Biotechnology) to identify macrophages and RAGE-expressing cells, respectively. Briefly, liver sections were dewaxed in 2 changes of xylene and rehydrated through graded alcohols. Antigen retrieval was performed by microwaving slides at 100°C for 30 minutes in 10 mM sodium citrate, pH 6.0, followed by peroxidase block, avidin and biotin block, and block of nonspecific binding sites with appropriate serum diluted in PBS, according to the manufacturer’s instruction. The primary antibody was applied overnight at 4°C in a humidified chamber, followed by biotin-conjugated secondary antibody and streptavidin-conjugated horseradish peroxidase, according to the manufacturer’s instructions (ABC; Vector Laboratories). Avidin-biotin conjugates were visualized using a peroxidase substrate kit (Vector Laboratories). Dehydrated sections were mounted using Permount mounting media (MilliporeSigma). The positive areas were captured using the NIS-Elements software (Nikon) attached to a Nikon microscope mounted with a Nikon DXM 1200F camera (Nikon). Images with uniform settings of magnification, light, and exposure time were used for quantitative image analysis.

For co-IHC, 5 μm sections of FFPE human liver core biopsies were used following the standard ImmPRESS Duet Double Staining Polymer Kit (Vector Laboratories) protocol and using primary antibodies for RAGE (1:100 dilution, sc-365154, Santa Cruz Biotechnology) and CD-68 (1:200 dilution, ab125212, Abcam) to identify macrophages and RAGE-expressing cells, respectively. Briefly, liver sections were dewaxed in 2 changes of xylene and rehydrated through graded alcohols. Antigen retrieval was performed by microwaving slides at 100°C for 30 minutes in 10 mM sodium citrate, pH 6.0, followed by BLOXALL block, avidin and biotin block, and block of nonspecific binding sites with normal horse serum, according to the manufacturer’s instruction. The primary antibody was applied overnight at 4°C in a humidified chamber, followed by ImmPRESS Duet Reagent, and ImmPACT DAB EqV Substrate (Vector Laboratories), according to the manufacturer’s instructions. Nuclei were counterstained using Gill’s Hematoxylin solution (MilliporeSigma), and sections were dehydrated and mounted using Permount mounting media (MilliporeSigma). The positive areas were captured using the NIS-Elements software (Nikon) attached to a Nikon microscope mounted with a Nikon DXM 1200F camera. Images with uniform settings of magnification, light, and exposure time were used for quantitative image analysis.

For co-IF, frozen liver tissues were cut into 7 μm sections and fixed with 3% paraformaldehyde for 20 minutes; this quenches fluorescence from EGFP. Samples were permeabilized with 0.2 % triton X-100 (MilliporeSigma) in phosphate-buffered saline (PBS) (ThermoScientific) for 5 minutes and blocked in 5% normal goat serum + 5% glycerol in PBS for 1 hour at room temperature. Samples were then incubated overnight at 4°C with rat anti-F4/80 (1:200; 14-4801-82, eBiosciences) or mouse anti-RAGE (1:100 dilution; sc-365154, Santa Cruz Biotechnology). Nuclei were counterstained with DAPI (ThermoScientific) at a 1:10,000 concentration for 5 minutes. Slides were mounted in ProLong Glass Antifade Mountant (P36982, Invitrogen). Images were acquired using an LSM980 confocal microscope (Zeiss). Colocalization was assessed by selecting 5 random fields per slide and calculating a Pearson’s correlation coefficient for the intensity of the red and green channels using the JACoP plugin in ImageJ (NIH) (48).

### IHL isolation.

IHLs were isolated from equal amounts of liver tissue within each dietary group. Liver tissue was dissociated with the mouse liver dissociation kit (Miltenyi Biotec) and the gentleMACS Dissociator (Miltenyi Biotec) following manufacturer protocols. The enzymatically digested liver was passed through a 70 μm filter (Falcon 352340; BD Biosciences) to remove debris and cell clusters. The filtered dissociate was centrifuged at 300*g* for 5 minutes. The cells were resuspended in 5 mL 25% Percoll (MilliporeSigma) in a 15 mL falcon tube, and 2 mL of 50% Percoll was slowly laid on the bottom of the same tube with a glass Pasteur pipette. The tube was centrifuged at 300*g*, with brakes removed, for 20 minutes at 4°C. The tube was carefully removed from the centrifuge, 2–3 mL of debris was removed from the top of the gradient, and using a glass Pasteur pipette, cells from the interface were recovered. Percoll was removed by transferring the recovered cells to a 50 mL Falcon tube, bringing the volume to 40 mL with serum-free RPMI (ThermoScientific) and spinning at 400*g* for 10 minutes at 4°C. The supernatant was removed, and cells were resuspended in PBS containing 0.5% BSA and 2 mM EDTA. Cells were counted in 2 quadrants in a hemocytometer and subsequently aliquoted for flow cytometry, CyTOF, and gene expression analysis. For gene expression analysis, cells were homogenized in TRIzol reagent (Invitrogen), and RNA was extracted using the RNeasy mini prep kit (QIAGEN). qPCR and multiplex gene expression analyses were performed as described below.

### qPCR.

RNA was extracted from IHL and BMDM by suspending in TRIzol reagent. The aqueous phase after the addition of 200 μL of chloroform and centrifugation was transferred to new Eppendorf tubes. After the addition of an equal volume of ethanol, the mixture was applied to the RNA isolation column, and RNA was extracted according to the manufacturer’s instructions. An in-column DNase digestion step was also performed. Isolated RNA quantity and quality were assessed with a NanoDrop ND1000 (Thermo Fisher Scientific). Reverse transcription was performed with the iScript cDNA synthesis kit (Bio-Rad). qPCR reactions were run on QuantStudio 6 Flex Real-Time PCR System using the Applied Biosystems Power SYBR Green PCR Master Mix (Applied Biosystems). Murine primer for *Ager* is provided in Supplemental Table 1C. All data are expressed as fold change relative to control (respective vehicle-treated control groups) as ΔΔCt using *18s* and hypoxanthine-guanine (*Hprt*) as housekeeping genes.

### NanoString panel.

NanoString analysis was performed with RNA isolated from IHLs of mice isolated as described above. RNA expression of 581 genes was determined (nCounter Mouse Immunology panel, NanoString Technologies). After hybridization, transcripts were quantified (nCounter NanoString Technologies), and results were normalized in the nSolver Analysis Software (NanoString Technologies) by the geometric mean of 10 housekeeping genes and 6 positive controls. Data were analyzed by ROSALIND (https://rosalind.onramp.bio/), with a HyperScale architecture developed by OnRamp BioInformatics Inc. Read distribution percentages, violin plots, identity heatmaps, and sample MDS plots were generated as part of the QC step. The limma R library was used to calculate fold changes and *P* values and perform optional covariate correction. Genes found different between the groups were reported, along with their magnitude of change (log_2_ scale) and their level of significance (FDR < 5%). Canonical pathway analysis was performed using IPA software (Ingenuity Systems). Biological functions and disease information within the IPA software were used to investigate the canonical pathways of interest. A stringent cutoff for *Z* scores of greater than 2 or less than −2 was used to consider activation and inhibition, respectively, with significance cutoffs of *P* ≤ 0.05 unless otherwise specified.

### Biochemical assays.

Plasma ALT levels were measured using a commercial veterinary chemistry analyzer (VetScan 2; Abaxis). The change in ALT was expressed as percentage of control to normalize for batch effects across different cohorts of mice.

### Flow cytometry.

After isolation of IHLs, cells were incubated with FcR Blocking Reagent (Miltenyi Biotec) for 5 minutes at 4°C, followed by labeling with fluorochrome-conjugated specific antibodies (Supplemental Table 1A) for 10 minutes at room temperature in the dark. Cells were centrifuged at 400*g* for 6 minutes at room temperature. Cells were washed once with protein extraction buffer and were centrifuged for 5 minutes at 400*g* at 4°C. The supernatant was removed, and cells were either analyzed fresh or fixed with 1% paraformaldehyde for flow cytometry the next day. Flow cytometry was performed with the MACSQuant Analyzer 10 (Miltenyi Biotec). Data were analyzed and graphed in FlowJo software, version 10.6.1. Cell numbers are presented as cell count per gram of liver tissue used for IHL isolation.

### CyTOF analysis.

Isolated IHLs were used for CyTOF performed at the Immune Monitoring Core at Mayo Clinic. An antibody cocktail of the entire phenotyping panel was prepared as a master mix using specific cell surface marker antibodies either purchased directly from Fluidigm or from the designated manufacturers (Supplemental Table 1B). Custom conjugated antibodies were generated in house through the Mayo Clinic Hybridoma Core by using Maxpar X8 Ab labeling kits (Fluidigm) according to the manufacturer’s protocol. Samples were loaded onto a Helios CyTOF system (Fluidigm) and were acquired at a rate of 200–400 events per second. Data were collected as.FCS files using the CyTOF software (version 6.7.1014). After acquisition, intrafile signal drift was normalized to the acquired calibration bead signal by using the CyTOF software. Cleanup of cell debris and removal of doublets and dead cells were performed with Cytobank cloud-based platform (Beckman Coulter).

### RNA-Seq of human liver biopsies.

After approval by the Mayo Clinic IRB, written informed consent was obtained from all patients for participation in medical research, including histological assessment of liver biopsies (IHC and NAS) (49) and RNA-Seq. Specimens were obtained from patients who had undergone liver biopsy during bariatric surgery for medically complicated obesity or from patients with obesity undergoing resection of benign liver masses at Mayo Clinic. The diagnosis of NASH and histological categorization according to established criteria was chosen a priori as follows: (a) normal obese (patients with BMI > 35 kg/m^2^ and normal liver histology), (b) nonalcoholic fatty liver (patients with BMI > 35 kg/m^2^ and presence of at least 5% macrovesicular steatosis on histology), and (c) advanced NASH (patients with BMI > 35 kg/m^2^ and presence of NASH on histology characterized by steatosis, at least grade 1 lobular, and/or portal inflammation as well as stage 2–4 fibrosis). Subjects with secondary causes of steatohepatitis and other chronic liver diseases were excluded. IHC was performed as described above with a primary antibody for RAGE (1:100 dilution, sc-365154, Santa Cruz Biotechnology). Total RNA was isolated from the snap-frozen liver biopsy samples (RNeasy Plus kit, Qiagen), and quality was initially assessed using Qubit fluorometry (Invitrogen) and the Agilent Fragment Analyzer. cDNA libraries were prepared using 300 ng of total RNA according to the manufacturer’s instructions for the TruSeq RNA Sample Prep Kit v2 (Illumina). The concentration and size distribution of the completed libraries were determined using an Agilent Bioanalyzer DNA 1000 chip and Qubit fluorometry (Invitrogen). Libraries were sequenced at approximately 50 million read pairs per lane following Illumina’s standard protocol using the Illumina cBot and HiSeq 3000/4000 PE Cluster Kit. The flow cells were sequenced as 100 X 2 paired-end reads on an Illumina HiSeq 4000 using HiSeq 3000/4000 sequencing kit and HD 3.3.52 collection software. Base calling was performed using Illumina’s RTA version 2.7.3. The RNA-Seq samples were sequenced by the Mayo Medical Genome Facility and processed through Mayo Clinic’s MAP-RSeq V2 software (50), a comprehensive RNA-Seq workflow for Illumina paired-end reads. Ensemble’s Homo sapiens GRCh38.78 was used for the reference genome and transcriptome. Quality-control metrics from RSeqQC were evaluated to ensure that raw expression values from each sample were reliable and could be collectively used for differential expression analysis (51). Genes with an average of ≥ 25 reads were kept for differential expression analysis. The R package edgeR was used to identify which genes were statistically differentially expressed from the group comparisons (52). Although reads per kilobase of transcript per million reads mapped (RPKM) are used to measure gene expression levels, it represents the relative abundance of a transcript among all sequenced transcripts and, therefore, depends on the RNA composition in each individual sample (53). Therefore, plotting RPKM values cannot be directly used to compare relative gene expression across samples. Instead, data are summarized as average fold change in our group of interest, patients with NASH with advanced fibrosis, relative to the other comparator groups.

### Macrophage–T cell in vitro crosstalk.

BMDM were isolated from hind legs of C57BL/6J mice as previously described (54). Once euthanized, the mouse was sprayed with 70% ethanol, and the skin, hip and ankle joints were cut open using sterile scissors to expose and remove the hind legs. The leg muscle and epiphyses were removed, and BM was flushed out onto a petri dish using a syringe and 25 gauge needle containing BMDM media, consisting of RPMI-1640 supplemented with 20% L929 cell-conditioned medium (LCM), 10% low endotoxin (LE) FBS, penicillin (100 units/mL), and streptomycin (100 μg/mL) (all from ThermoScientific). The flushed media containing BM was drawn through a 25 gauge needle 4–5 times to remove clumps. BM cells were plated onto 150 mm petri dishes (BD Falcon) and incubated at 37°C, 5% CO_2_. BMDM media was changed every 2 days — on days 3 and 5 — and BMDMs were dissociated with Accutase and used in experiments on day 7. BMDMs from WT or RAGE-MKO mice were primed in plain RPMI for 2 hours, after which they were treated with mouse rS100A11 (50227-M07E, Sinobiologicals) or control for 6 hours in fresh RPMI, supplemented with 1% fatty acid free BSA. After 6 hours, the cell supernatant was collected and centrifuged at 300*g* for 5 minutes at 4°C to remove debris and stored at –20°C. Separately, T cells were isolated from the spleen of a WT mouse and enriched for CD8^+^ T cells using the CD8a^+^ T Cell Isolation Kit (Miltenyi Biotec) and plated at a density of 1 × 10^5^ cells per well of a 96-well plate. Cells were suspended in 100 μL T cell media, which consists of RPMI + 10% low endotoxin-FBS and 1% penicillin (100 units/mL) and streptomycin (100 μg/mL). These wells were treated with 100 μL supernatant collected from treated BMDMs along with 2.5 μL of CD3/CD28 (Dynabeads, Thermo Fisher Scientific) for 48 hours. Supernatant from these wells was collected, centrifuged at 300*g* for 5 minutes at 4°C to remove debris, and 50 μL of the media was used for quantification for IFN-γ quantification by ELISA (Invitrogen, BMS606-2) following manufacturer protocol. Data were normalized as percentage of maximum IFN-γ release within each experimental run.

### Statistics.

Data are presented as median ± IQR or mean ± SEM as appropriate. A nonparametric test (Mann-Whitney *U*) was used for comparing groups, and *P* values were adjusted by the Benjamini-Hochberg method for multiple comparisons. Statistical analyses were performed in GraphPad Prism version 6.00 for Windows (GraphPad Software). Each dot on dot plots represents an independent biological replicate. An adjusted *P* < 0.05 was considered significant.

### Study approval.

All animal procedures were performed in an AAALAC-accredited facility in accordance with the *Guide for the Care and Use of Laboratory Animals* (National Academies Press, 2011) and approved by the Mayo Clinic IACUC. After approval by the Mayo Clinic IRB, written informed consent was obtained from all patients for participation in medical research, including histological assessment of liver biopsies (IHC and NAS) (49) and RNA-Seq.

### Data availability.

Values for all data points in graphs are reported in the Supporting Data Values file. Data from the NanoString gene (mRNA) expression profiles have been submitted to the Gene Expression Omnibus database and are available under accession no. GSE243295.

## Author contributions

GP contributed to the study concept and design; acquisition, analysis, and interpretation of data; and drafting of the manuscript. ASM, NG, LHK, PVD, GSS, and AK contributed to acquisition, analysis, and interpretation of data. EL, PVD, HSKL, GWM, PH, and KDP contributed to acquisition analysis and interpretation of data as well as technical support. MC and SII contributed to interpretation of data and critical revision of the manuscript for important intellectual content. HM contributed to study concept and design; analysis and interpretation of data; critical revision of the manuscript for important intellectual content; funding acquisition; administrative, material, and technical support; and study supervision.

## Supplementary Material

Supplemental data

Supporting data values

## Version 1 01/04/2024

In-Press Preview

## Version 2 02/08/2024

Electronic publication
