# Supplementary material for: Macrophage RAGE activation is proinflammatory in NASH
Source: JCI Insight. 2024 Feb 8;9(3):e169138. doi: 10.1172/jci.insight.169138 (PMC10967390; doi:10.1172/jci.insight.169138)
Supplement: Supplemental data [file jciinsight-9-169138-s312.pdf]

Supplementary Table 1A

Flow Cytometry Antibodies		
**Target**	**Vendor**	**Fluourophore**
Live/Dead	Miltenyi Biotec	REA-Viogreen
CD45	Miltenyi Biotec	REA737-Vioblue
F4/80	Miltenyi Biotec	REA126-PE
CD11b	Miltenyi Biotec	REA592-PerCP Vio700
RAGE	Santa Cruz Biotechnology	Mouse-APC
CD3	Miltenyi Biotec	Rat-APC Vio770
CD4	Miltenyi Biotec	REA-PerCP Vio700
CD8	BioLegend	Rat-BV 421

Supplementary Table 1B

CyTOF antibodies							
**No.**	**Sp.**	**Label**	**Target**	**Clone**	**Localization**	**Vendor**	**Catalog number**
1	Ms	089Y	CD45	30-F11	Surface		
2	Ms	106Cd	CD44	IM7	Surface	Biolegend	103051
3	Ms	110Cd	CXCR6	221002	Surface	R&D	MAB2145-100
4	Ms	111Cd	IgM	RMM-1	Surface	Biolegend	406527
5	Ms	112Cd	Ly6G	1A8	Surface	Biolegend	127637
6	Ms	113Cd	F4/80	BM8	Surface	Biolegend	123101
7	Ms	116Cd	CD11c	N418	Surface		
8	Ms	141Pr	Granzyme B	QA16A02	Intracellular/Nuclear		
9	Ms	142Nd	Eomes	Dan11mag	Intracellular/Nuclear		
10	Ms	143Nd	TCRb	H57-597	Surface		
11	Ms	144Nd	Tcf1	812145	Intracellular/Nuclear		
12	Ms	145Nd	CD69	H1.2F3	Surface		
13	Ms	146Nd	Gata3	TWAJ	Intracellular/Nuclear		
14	Ms	148Nd	ROR gamma (t)	B2D	Intracellular/Nuclear		
15	Ms	149Sm	Tim4	370900	Surface		
16	Ms	150Nd	RAGE	697023	Surface	R&D	MAB11795
17	Ms	151Eu	IRF5	903430	Intracellular/Nuclear	R&D	MAB8447
18	Ms	152Sm	CD3e	145-2C11	Surface		
19	Ms	153Eu	IgD	11-26c.2a	Surface		
20	Ms	154Sm	BATF	D7C5	Intracellular/Nuclear		
21	Ms	155Gd	Tbet	4B10	Intracellular/Nuclear		
22	Ms	156Gd	CCR2	475301	Surface		
23	Ms	158Gd	FoxP3	FJK-16s	Intracellular/Nuclear		
24	Ms	159Tb	CD279 (PD-1)	RMP1-30	Surface		
25	Ms	160Gd	CD62L	MEL14	Surface		
26	Ms	161Dy	Ki-67	B56	Intracellular/Nuclear		
27	Ms	163Dy	CD4	RM4-5	Surface		
28	Ms	164Dy	CX3CR1	SA011F10	Surface		
29	Ms	165Ho	TCR g/d	GL3	Surface		
30	Ms	166Er	CD19	6D5	Surface		
31	Ms	167Er	TREM2	237920	Surface	R&D	MAB17291-100
32	Ms	168Er	CD8a	53-6.7	Surface		
33	Ms	169Tm	CD206 (MMR)	C068C2	Surface		
34	Ms	170Er	CD161 (NK1.1)	PK136	Surface		
35	Ms	171Yb	CD11b	M1/70	Surface		
36	Ms	173Yb	IFNAR	MAR1-5A3	Surface	Biolegend	127302
37	Ms	174Yb	IRF7	SC0617	Intracellular/Nuclear	Fisher scientific	NBP267634
38	Ms	175Lu	Ly6C	HK1.3	Surface		
39	Ms	175Lu	iNos (NOS2)	CXNFT	Intracellular/Nuclear		
40	Ms	176Yb	CD45R (B220)	RA3-6B2	Surface		
41	Ms	209Bi	I-A/I-E	M5/114.15.1	Surface		

Supplementary Table 1C

Mouse AGER primer

Forward primer	ACAGGCTCTGTGGGTGAGTCT
Reverse primer	CTGACTGATTCAGCTCTGCAC

Supplementary Table 2

FFC vs CD		
**Transcript**	**log2FoldChange**	**Adjusted p-value**
Irf3	-1.91557922303003	0.01164190678107
Il17f	-1.67846385312494	0.04978409791617
Jak3	-1.45190114933885	0.02303334662423
Notch1	-1.44092586313036	0.02793889178648
Mapk11	-1.27692060108747	0.04978409791617
Prdm1	1.22343174833689	0.03166392234792
Runx3	1.38639166320690	0.02169540006044
Itga4	1.47466592087780	0.04978409791617
Ccl3	1.52643161579386	0.04978409791617
Tlr1	1.66115563898585	0.03863995969144
Clec5a	1.71496150109695	0.03166392234792
Cd8b1	1.83946103441762	0.03166392234792
Ccl22	2.02143849660410	0.02169540006044
Slamf7	2.04192614966686	0.02303334662423
Itgax	2.07517145118005	0.00993922898369
Ptafr	2.18451955974928	0.02169540006044
Clec4e	2.27188232451764	0.00993922898369
Il12b	2.35841272532415	0.02624131009093
Ccr2	2.44099952728534	0.00993922898369
Il1rn	2.56116575792370	0.00993922898369
Cx3cr1	2.62322973682260	0.00993922898369
Trem2	2.72157118662470	0.03166392234792
Ccl9	2.87936355297890	0.00993922898369
Pdcd1	3.40473237607752	0.02169540006044

Common
**Transcript**
Irf3
Il17f
Jak3
Runx3
Il12b
Pdcd1

TTP vs Vehicle		
**Transcript**	**log2FoldChange**	**Adjusted p-value**
Pdcd1	-2.70083986395017	0.00831862416614
Tigit	-2.12970765566487	0.02458624400983
Il12b	-1.91638537697839	0.01895217688265
Il21	-1.44973467095734	0.01686754173649
Ebi3	-1.43634608365590	0.01957767158190
Runx3	-1.34330418849813	0.02846856304294
Tnfrsf8	-1.30752930293708	0.00202822278092
Batf	-1.03536702727563	0.01462997948220

Spn	-0.92436175069813	0.01963291598104
Ccr9	-0.75736088944014	0.04649277984736
Ikbkap	0.61680255884382	0.04606970009190
Jak3	0.70710425154731	0.04346605012743
C6	0.77932603316107	0.04669719625760
Itga2b	0.87563259688668	0.03675969341578
Tirap	0.89862436540173	0.02078235166748
Ccr4	0.95091478225381	0.02556026576102
Ctsg	0.95091478225381	0.02556026576102
Cfh	0.97616748319622	0.03083765062697
C2	1.01812001104347	0.01137742485369
Ccl25	1.04380240942263	0.03887481531857
Cxcl12	1.12620569483799	0.02085725092623
Serping1	1.12847180008940	0.04618347515817
Irf3	1.14558934544881	0.00532987406691
C9	1.15006896599936	0.04260734933925
Il17f	1.16186821599424	0.03266606955581
Cd209g	1.23577454437726	0.02074682743569
C4a	1.24830595239809	0.01219818328162
C8a	1.25330659651595	0.01132475712725
Hc	1.25722609349807	0.03135396691900
Icam4	1.26770949043196	0.01011855625046
C4bp	1.28726282205908	0.01756956951470
Cd28	1.30232707275700	0.02638246229733
C3	1.30468415832763	0.01903752042045
Ncam1	1.37872472948204	0.00248057605913
C1s	1.38944411007308	0.00921107018050
Il23a	1.40419737525004	0.03316471069197
Src	1.41508641153450	0.00466933574076
Il25	1.41680607570068	0.02466581453118
Cxcl3	1.44468566766554	0.00907808894183
Defb1	1.60251091429003	0.02502164908419
Il17re	1.60433233999885	0.00423830520874
Ccr10	1.67124530870240	0.00208392758496
C8b	1.90661555536523	0.01586340747850
Hamp	1.95797186063560	0.00120142640956
Tgfb2	2.08618655324588	0.01660832391571

FFC vs CD		
**Transcript**	**log2FoldChange**	**p-value**
Clec5a	-2.42	0.0056425
Il2ra	-2.37	0.00933344
Gata3	-2.33	0.00297017
Il12b	-2.22	0.00910749
Cxcr2	-2.06	0.00053158
Camp	-1.98	0.00475722
Trem1	-1.96	0.00090398
Il18rap	-1.94	0.00357194
Clec4e	-1.89	0.000597
Thy1	-1.79	0.00587723
Il1r2	-1.71	0.00821676
Cebpb	-1.67	0.0037694
Tnf	-1.65	0.00036085
Bcl3	-1.65	0.00398876
Map4k1	-1.62	0.00087404
Ifitm1	-1.61	0.00217902
Ptgs2	-1.54	0.00334
Gpr183	-1.5	0.00263025
Card9	-1.43	0.00582906
Alas1	-1.42	0.00734253
Ikzf1	-1.37	0.00429544
Tcf7	-1.36	0.0038067
Nfatc2	-1.28	0.0035939
Xcr1	-1.26	0.00950485
Csf3r	-1.23	0.00474731
Bst1	-1.23	0.00465035
H2-Ob	-1.15	0.00883139
Ikzf3	-1.13	0.00958845
Csf2rb	-1.12	0.00075657
Ccl22	-1.07	0.0069127
Notch2	-1.05	0.00043886
Prdm1	-1	0.00278484
Relb	-0.98	0.00306336
Tlr9	-0.96	0.0025249
Stat5a	-0.94	0.00066533
Nfkb2	-0.87	0.00828366
Runx1	-0.85	0.00742405
Il6ra	-0.85	0.00180312
Irak2	-0.82	0.00355641
Cmklr1	-0.76	0.00356644
Il2rg	-0.49	0.00802569
Ddx58	0.59	0.00751043
Cd1d1	0.67	0.00979999
Casp1	0.8	0.00581094
Plau	0.84	0.00619039

Ifit2	0.87	0.00404409
Lilra5	1.12	0.00862765
Irgm1	1.15	0.00124713
Irf1	1.18	0.00875172
Tcf4	1.27	0.00458802
Tnfsf10	1.53	0.00466106
Ly96	1.55	0.00889545
Cd34	1.75	0.00013003
Ets1	1.93	0.00789056
Cd81	1.98	0.00291383
App	2.1	0.00897349
Fcgr4	2.14	0.00989835
Cfh	2.25	0.00459037
Cd55	2.56	0.00926235
Pecam1	2.82	0.00161973
Clu	3.2	0.00888944
Fcgr2b	4.03	0.00801374

Supplementary Table 3

FFC vs CD			
**Ingenuity Canonical Pathways**	**-log(p-value)**	**Ratio**	**z-score**
Calcium-induced T Lymphocyte Apoptosis	3.02	0.0656	4
Th1 Pathway	77.9	0.624	2.832
Role of NFAT in Regulation of the Immune Response	14.8	0.114	2.469
Crosstalk between Dendritic Cells and Natural Killer Cells	55.6	0.662	2.343
Huntington's Disease Signaling	3.22	0.0664	2.333
Cyclins and Cell Cycle Regulation	2.11	0.0854	2.236
Systemic Lupus Erythematosus In T Cell Signaling Pathway	14.5	0.108	2.16
ICOS-ICOSL Signaling in T Helper Cells	15.4	0.13	2.121
FAK Signaling	30.2	0.111	2.085
Complement System	32.1	0.758	2
Apelin Cardiac Fibroblast Signaling Pathway	2.47	0.174	2
Cell Cycle: G1/S Checkpoint Regulation	3.38	0.123	-2
IL-15 Production	14.9	0.214	-2.041
MSP-RON Signaling In Macrophages Pathway	27.6	0.33	-2.058
Oncostatin M Signaling	7.88	0.262	-2.111
PDGF Signaling	4.6	0.128	-2.111
ATM Signaling	2.8	0.0918	-2.236
Endothelin-1 Signaling	2.69	0.0703	-2.309
Role of NFAT in Cardiac Hypertrophy	2.11	0.0599	-2.309
Mouse Embryonic Stem Cell Pluripotency	7.69	0.157	-2.324
SPINK1 General Cancer Pathway	4.67	0.161	-2.333
IL-17A Signaling in Airway Cells	22.2	0.397	-2.4
Role of NANOG in Mammalian Embryonic Stem Cell Pluripotency	3.35	0.0932	-2.53
GADD45 Signaling	13.8	0.305	-2.668
Senescence Pathway	12.1	0.12	-2.959

Common
**Ingenuity Canonical Pathways**
Calcium-induced T Lymphocyte Apoptosis
Role of NFAT in Regulation of the Immune Response
Crosstalk between Dendritic Cells and Natural Killer Cells
Senescence Pathway

TTP vs Vehicle			
**Ingenuity Canonical Pathways**	**-log(p-value)**	**Ratio**	**z-score**
Phagosome Formation	19.5	0.102	-3.101
Breast Cancer Regulation by Stathmin1	5.01	0.0616	-2.744
Calcium-induced T Lymphocyte Apoptosis	3.02	0.0656	-2.5
Inflammasome pathway	9.18	0.474	-2.333
CREB Signaling in Neurons	5.17	0.0619	-2.263
FcγRIIB Signaling in B Lymphocytes	1.51	0.0706	-2.236
Dendritic Cell Maturation	39.1	0.193	-2.183
Role of NFAT in Regulation of the Immune Response	14.8	0.114	-2.16
Coronavirus Pathogenesis Pathway	32.2	0.258	-2.064

Colorectal Cancer Metastasis Signaling	18.6	0.155	-2.058
Immunogenic Cell Death Signaling Pathway	24	0.354	-2.043
Crosstalk between Dendritic Cells and Natural Killer Cells	55.6	0.662	-2.03
Role of RIG1-like Receptors in Antiviral Innate Immunity	23.7	0.6	2.065
Sirtuin Signaling Pathway	2.84	0.0632	2.111
Activation of IRF by Cytosolic Pattern Recognition Receptors	30.5	0.537	2.268
Senescence Pathway	12.1	0.12	2.401

RAGE-MKO vs WT			
**Ingenuity Canonical Pathways**	**-log(p-value)**	**Ratio**	**z-score**
Pathogen Induced Cytokine Storm Signaling Pathway	44.6	0.125	-3.063
Th1 Pathway	37.4	0.244	-2.921
IL-12 Signaling and Production in Macrophages	34.7	0.141	-2.874
Th2 Pathway	32.5	0.204	-2.887
Macrophage Classical Activation Signaling Pathway	26.4	0.138	-3.272
Cardiac Hypertrophy Signaling (Enhanced)	24.8	0.0668	-3.053
Natural Killer Cell Signaling	24.6	0.128	-2.2
Cachexia Signaling Pathway	24.5	0.0842	-2.335
Multiple Sclerosis Signaling Pathway	23.1	0.112	-2.746
Systemic Lupus Erythematosus in B Cell Signaling Pathway	22.6	0.0534	-2.596
Role of Osteoblasts in Rheumatoid Arthritis Signaling Pathway	22.5	0.106	-2.353
Crosstalk between Dendritic Cells and Natural Killer Cells	22.2	0.207	-3.357
CDX Gastrointestinal Cancer Signaling Pathway	19.1	0.107	2.683
Wound Healing Signaling Pathway	18.3	0.0898	-2.711
NOD1/2 Signaling Pathway	17.3	0.106	-2.683
S100 Family Signaling Pathway	16.1	0.0425	-2.263
IL-33 Signaling Pathway	16.1	0.101	-2.524
IL-23 Signaling Pathway	15.5	0.261	-2.111
Activin Inhibin Signaling Pathway	14.8	0.0868	-2.065
IL-17A Signaling in Fibroblasts	14.8	0.161	-2.138
IL-17 Signaling	14.8	0.0952	-2.828
FAK Signaling	14.8	0.0345	-2
B Cell Activating Factor Signaling	14.2	0.256	-2.53
April Mediated Signaling	12.6	0.238	-2.333
Tumor Microenvironment Pathway	11.3	0.0798	-2.138
PPAR Signaling	10.9	0.112	2.714
Th17 Activation Pathway	10.5	0.0432	-2.982
RANK Signaling in Osteoclasts	10.3	0.12	-2.333
LXR/RXR Activation	8.69	0.0846	2.333
T Cell Receptor Signaling	8.53	0.0338	-2.837
Myelination Signaling Pathway	7.78	0.0446	-2.324
p38 MAPK Signaling	6.75	0.075	-2.333
Dendritic Cell Maturation	6.64	0.0302	-2.828
Adrenergic Receptor Signaling Pathway (Enhanced)	6.35	0.0502	2.53
G-Protein Coupled Receptor Signaling	6.15	0.0266	-3.3
NF-κB Signaling	5.54	0.028	-3
ICOS-ICOSL Signaling in T Helper Cells	5.45	0.0293	-3.742
Small Cell Lung Cancer Signaling	5.23	0.0714	-2.449
B Cell Receptor Signaling	4.32	0.0234	-2.887
CD27 Signaling in Lymphocytes	4.3	0.0877	-2
PI3K Signaling in B Lymphocytes	4.08	0.0235	-2.714
CREB Signaling in Neurons	3.93	0.0228	-2.496
WNT/Ca+ pathway	3.91	0.0725	-2
Systemic Lupus Erythematosus in T Cell Signaling Pathway	3.68	0.0215	-2.138
NFKBIE Signaling Pathway	2.88	0.0223	-2.53

CTLA4 Signaling in Cytotoxic T Lymphocytes	2.87	0.0196	2.309
Regulation of IL-2 Expression in Activated and Anergic T Lymphocytes	2.78	0.0216	-2.53
PKCθ Signaling in T Lymphocytes	2.68	0.0196	-2.53
Breast Cancer Regulation by Stathmin1	2.47	0.0184	-2.714
CD28 Signaling in T Helper Cells	2.41	0.0192	-2.646
IGF-1 Signaling	2.16	0.0374	2
Oxidative Phosphorylation	1.97	0.0328	2
Insulin Receptor Signaling	1.71	0.0274	2
OX40 Signaling Pathway	1.7	0.0167	-2.236
Calcium-induced T Lymphocyte Apoptosis	0.388	0.00862	-2

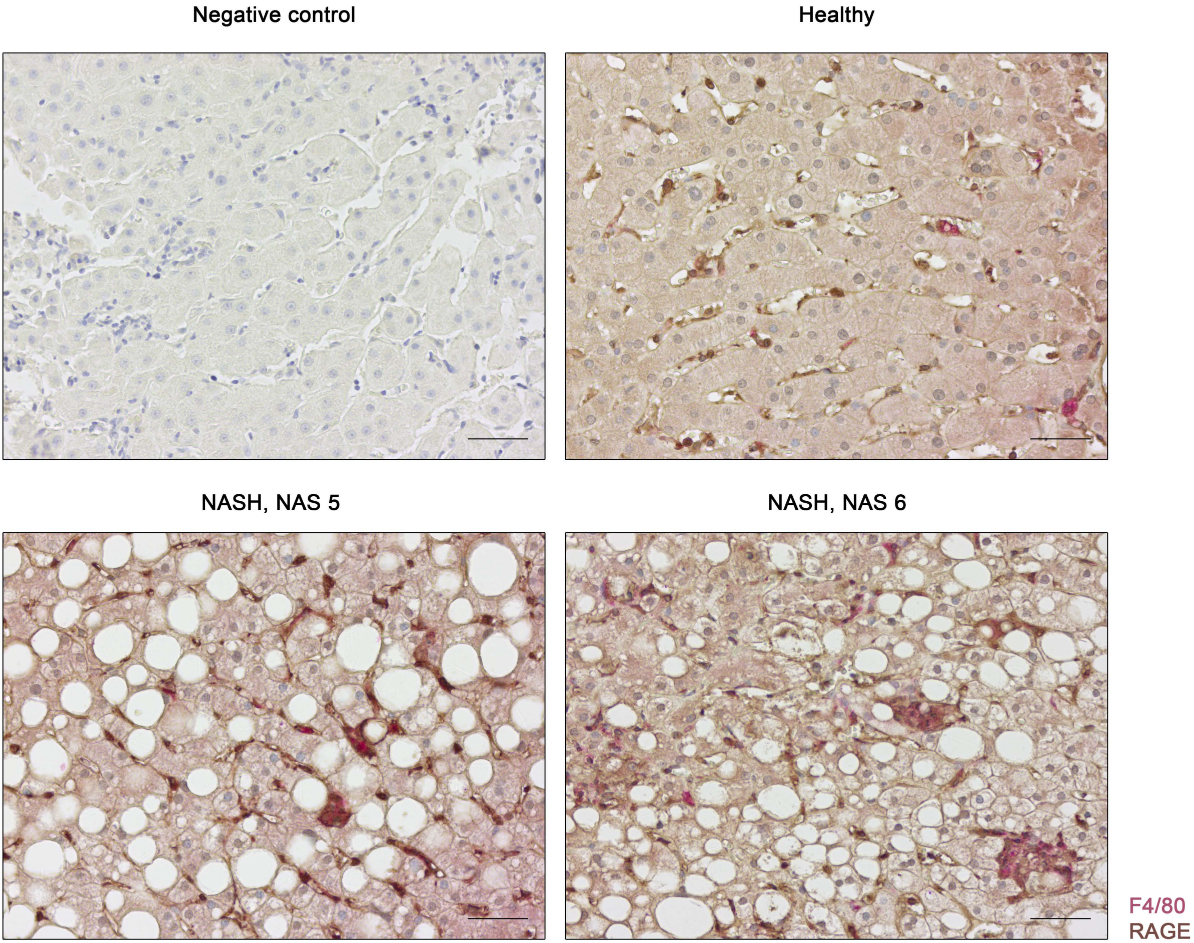

Supplementary Figure 1. RAGE expression is enriched on macrophages in human NASH.
Representative images of co-IHC for RAGE (brown) and F4/80 (pink) of livers from a healthy donor (n=5) and patients with NASH (n=5). The NAFLD activity score (NAS) is provided. A negative control stained with only the primary antibodies is shown. Scale bar=50 μm.

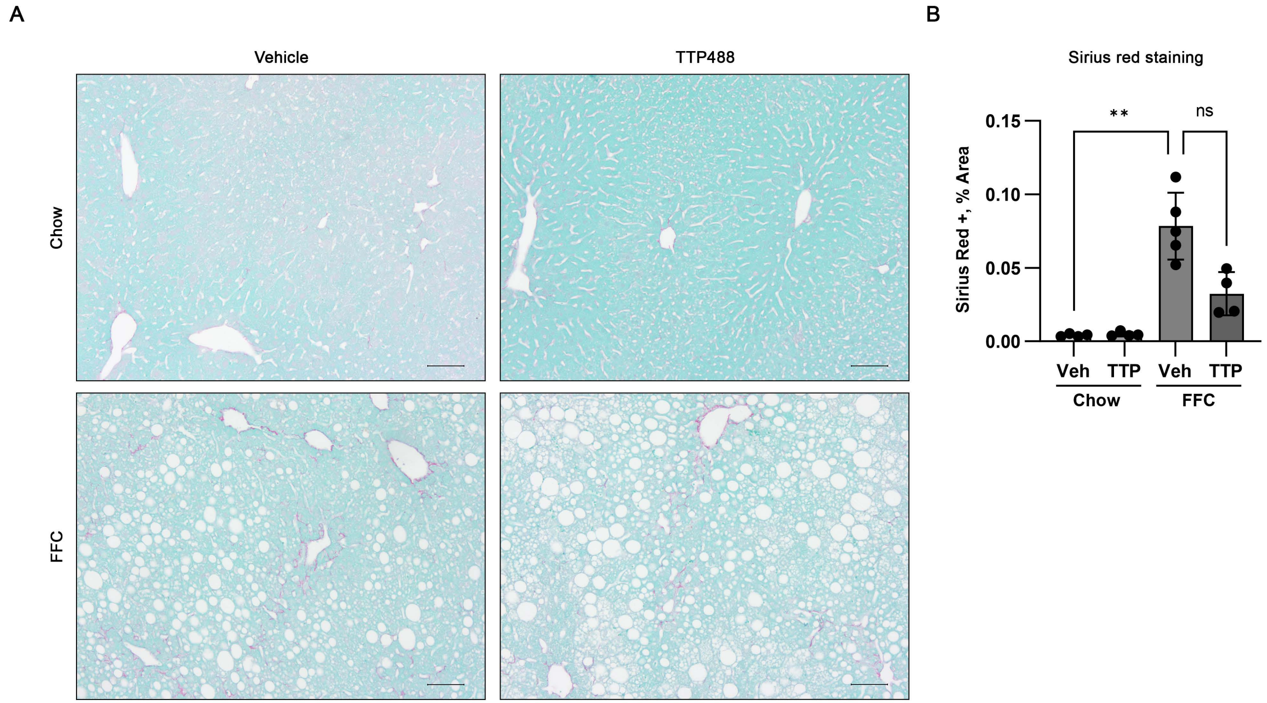

Supplementary Figure 2. Attenuation of FFC diet induced fibrosis with inhibition of macrophage RAGE signaling.
(A) Representative images of sirius red staining of livers from vehicle or TTP488 treated chow (n=4 each) and FFC mice (n=5 and n=4 respectively) demonstrating collagen staining in red, scale bar=50 μm, and (B) its quantification, $P<.01$. Mann-Whitney test was used for statistical analyses.

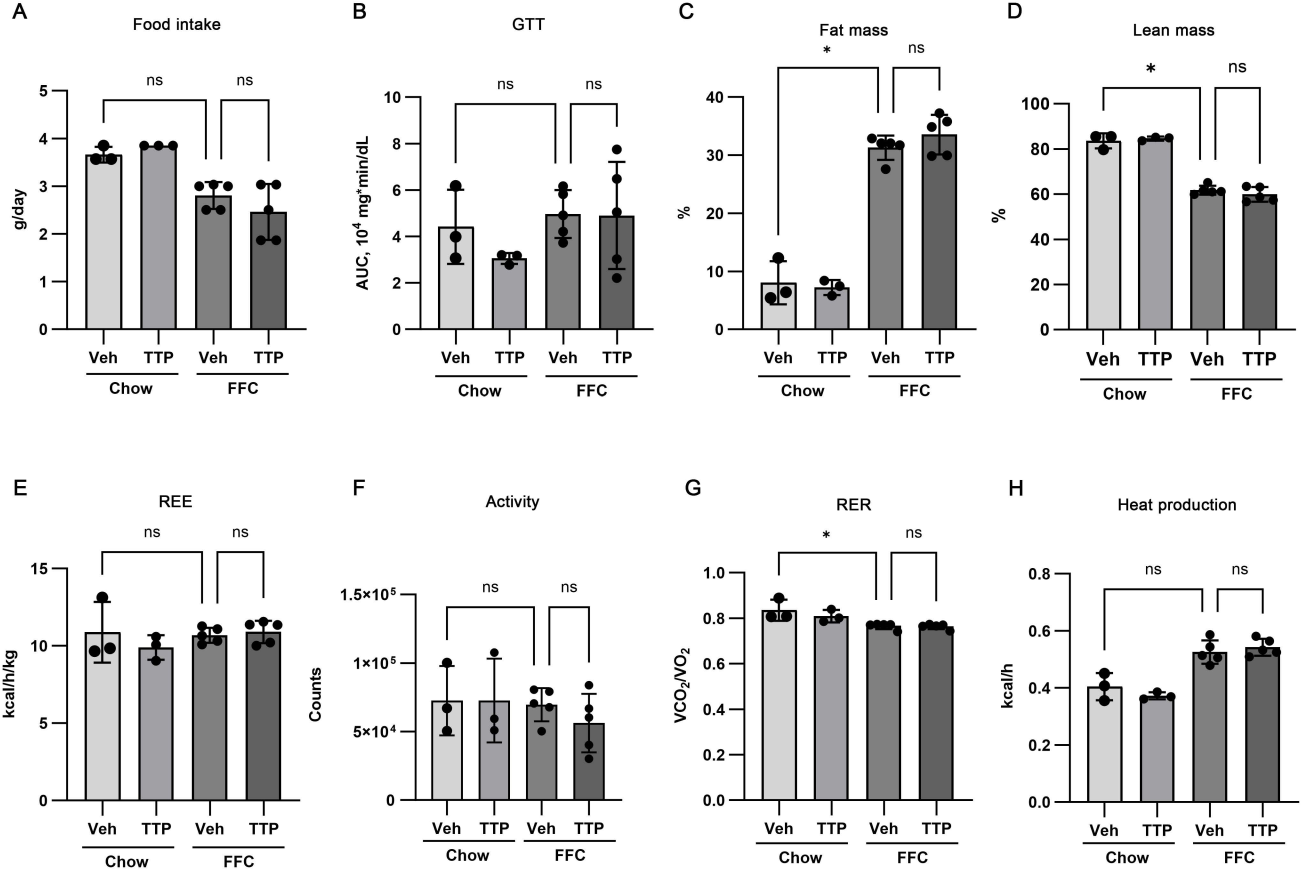

Supplementary Figure 3. Metabolic parameters of FFC mice are not different between vehicle and TTP488 treatment.
Among FFC mice, compared to vehicle treatment, TTP488 did not affect (A) food intake; (B) area under the curve during glucose tolerance test; (C) relative fat; (D) lean mass; (E) resting energy expenditure; (F) activity levels; (G) respiratory exchange ratio; (H) heat production (Chow-vehicle n=3, Chow-TTP n=3, FFC-vehicle n=5, FFC-TTP n=5 per group, $P<.05$); Mann-Whitney test was used for statistical analyses.

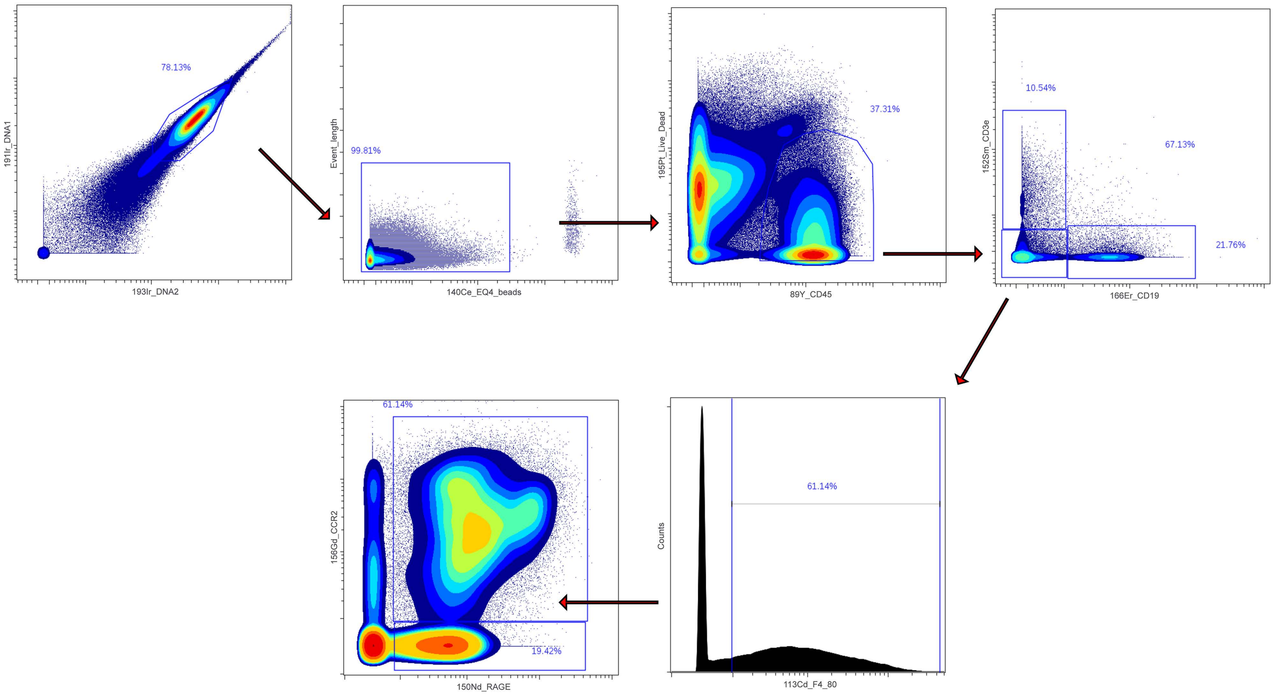

Supplementary Figure 4. Gating strategy by CyTOF to identify RAGE$^+$ macrophages as CD45$^+$CD3$^-$CD19$^-$F4/80$^+$RAGE$^+$ cells.

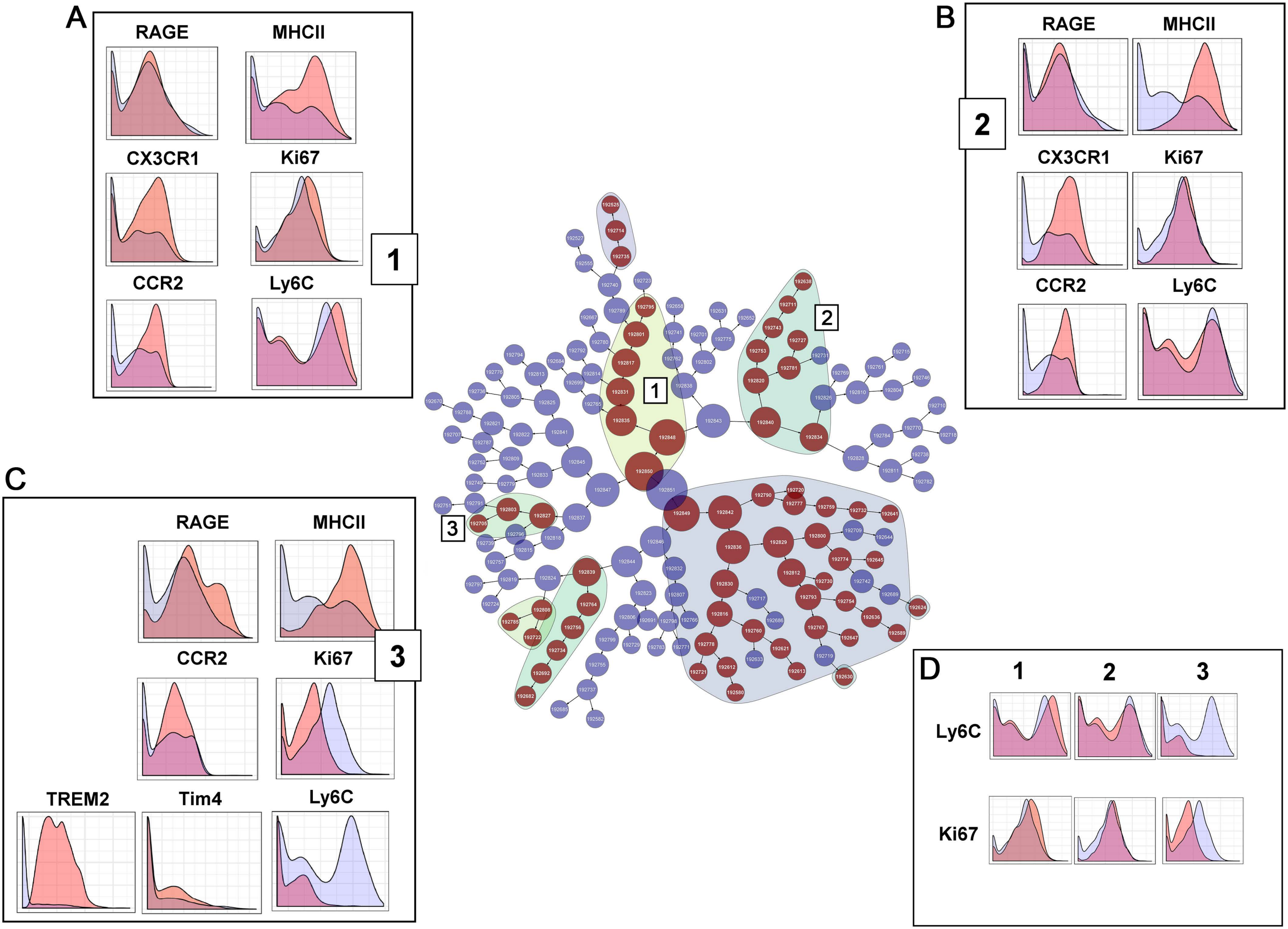

Supplementary Figure 5. Phenotypic characterization of the 3 RAGE enriched macrophage clusters by CITRUS analysis.
(A, B&C) Histograms depicting expression intensity of representative markers (orange shaded) compared to other clusters (blue) among RAGE expressing macrophages (1, 2&3, respectively) from vehicle and TTP488 treated chow and FFC mice. (D) Progressive decline in expression of Ly6C and Ki67 among the 3 clusters of RAGE expressing macrophages.

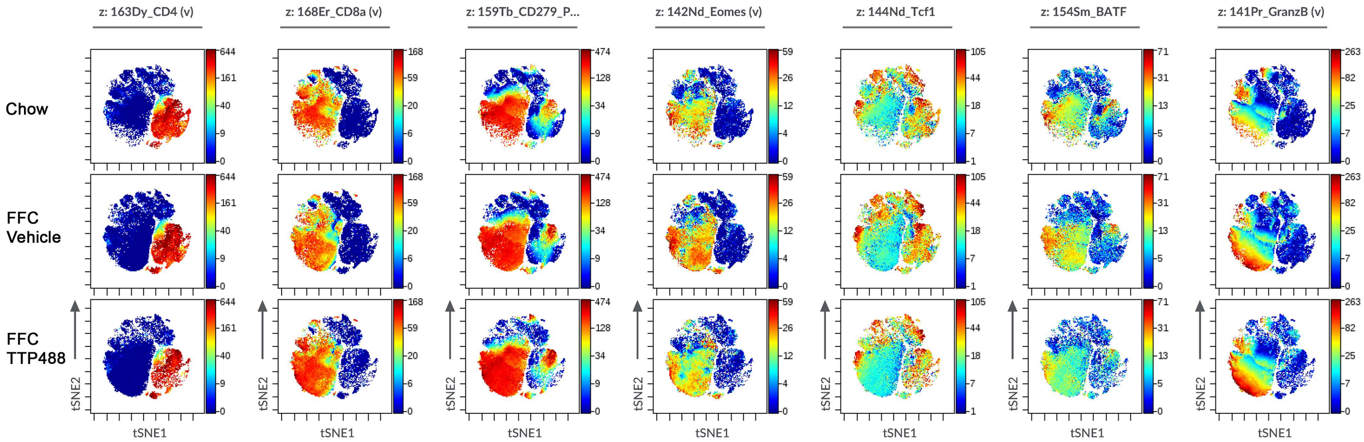

Supplementary Figure 6. Phenotypic characterization of T cells by CyTOF.
viSNE plots depicting expression of representative T cell markers among $CD3^{+}$ cells gated from IHLs isolated from chow and vehicle and TTP488 treated FFC mice.

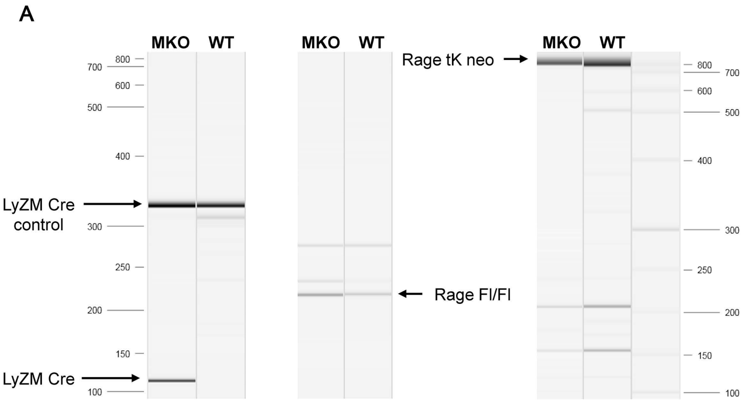

Genotype	Forward sequence	Reverse sequence
Rage fl/fl flox	atgccagcggggacagcagctag	ctagctgctggggcggcttctta
Rage tK-neo flox	cctcgaacaccgagcgaccctg	ctcagaagaactcgtcaagaaggc
LyzM Cre	gcggtctggcagtaaaaactatc	gtgaaacagcattgctgtcactt
LyzM Cre control	ctaggccacagaattgaaagatct	gtaggtggaaattctagcatcatcc

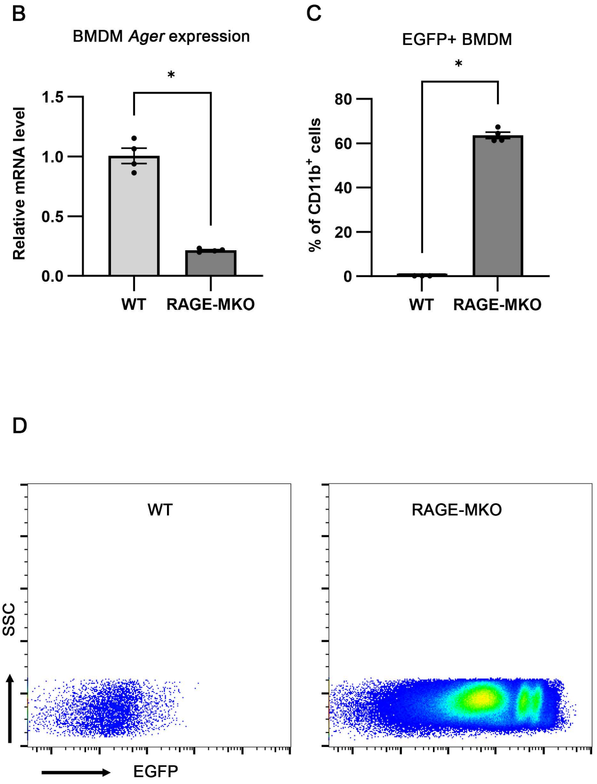

Supplementary Figure 7. Confirmation of myeloid-specific RAGE knockout.
(A) Representative QIAxcel capillary electrophoresis images of expression of LyZM, RAGE and the RAGE tK neo cassette; LyZM Cre control is included. (B) Relative mRNA expression of *Ager* in bone marrow derived macrophages (BMDM) from WT compared to RAGE-MKO mice (n=4 each), $P<.05$; (C) Comparison of EGFP expression on BMDM quantified by flow cytometry from WT (n=3) compared to RAGE-MKO mice (n=4) and expressed as percentage of all myeloid ($CD11b^+$) cells, $P<.05$ (D) Representative flow cytometry plots demonstrating EGFP expression on BMDM from WT compared to RAGE-MKO mice. Mann-Whitney test was used for statistical analyses.

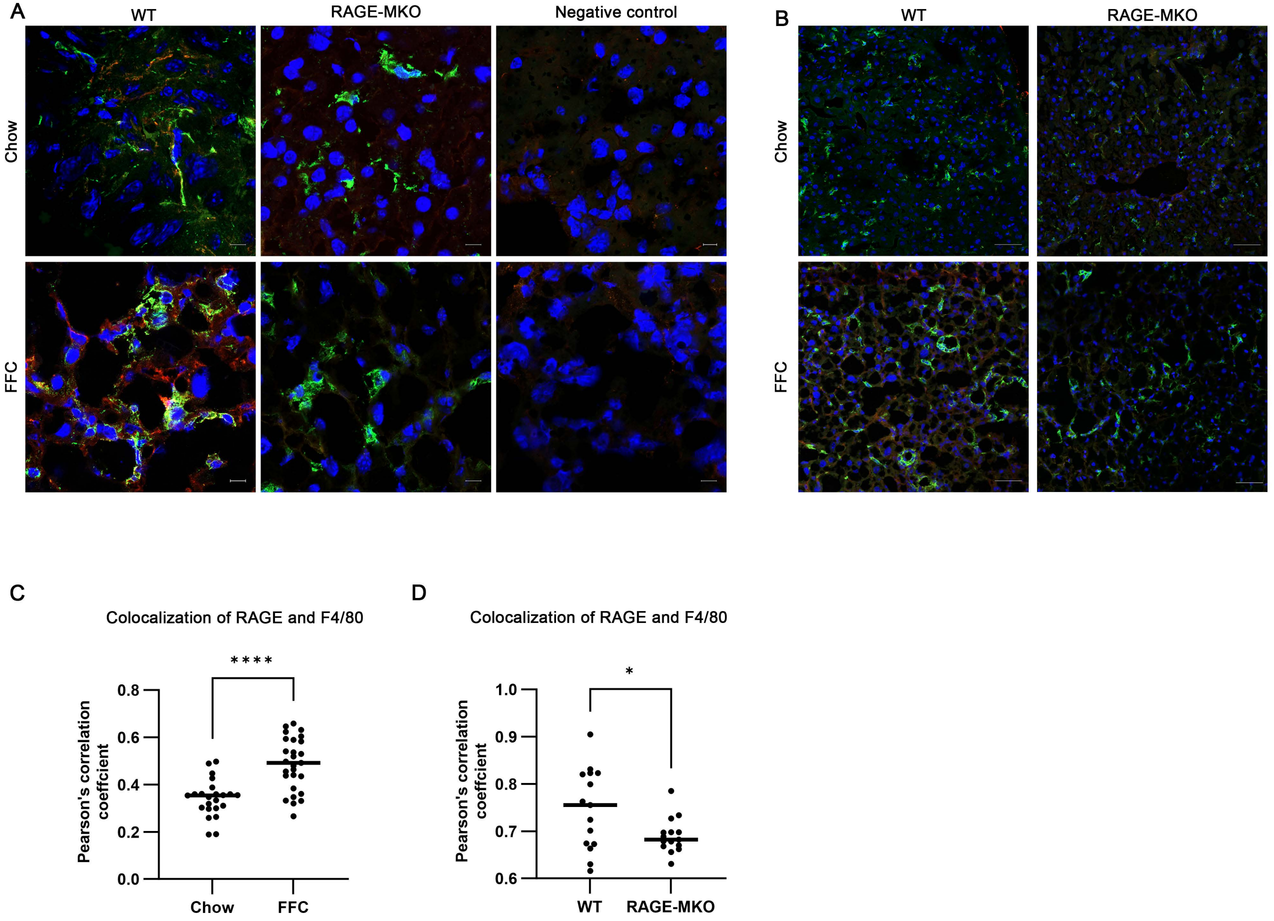

Supplementary Figure 8. Myeloid-specific RAGE knockout attenuates accumulation of RAGE+ macrophages in the liver.
(A) Representative co-IF images of liver cryosections stained for DAPI, F4/80 and RAGE from chow or FFC WT and RAGE-MKO mice. Negative controls are included. Scale bar=10 μm. (B) Representative co-IF images shown at lower magnification of liver cryosections stained for DAPI, F4/80 and RAGE from chow or FFC WT and RAGE-MKO mice. Scale bar=50 μm. (C) Quantification of RAGE co-localization with F4/80 from chow (n=5) and FFC (n=6) WT mice *P*<.05. (D) Quantification of RAGE co-localization with F4/80 from FFC WT (n=4) and RAGE-MKO (n=4) mice *P*<.05. Mann-Whitney test was used for statistical analyses.

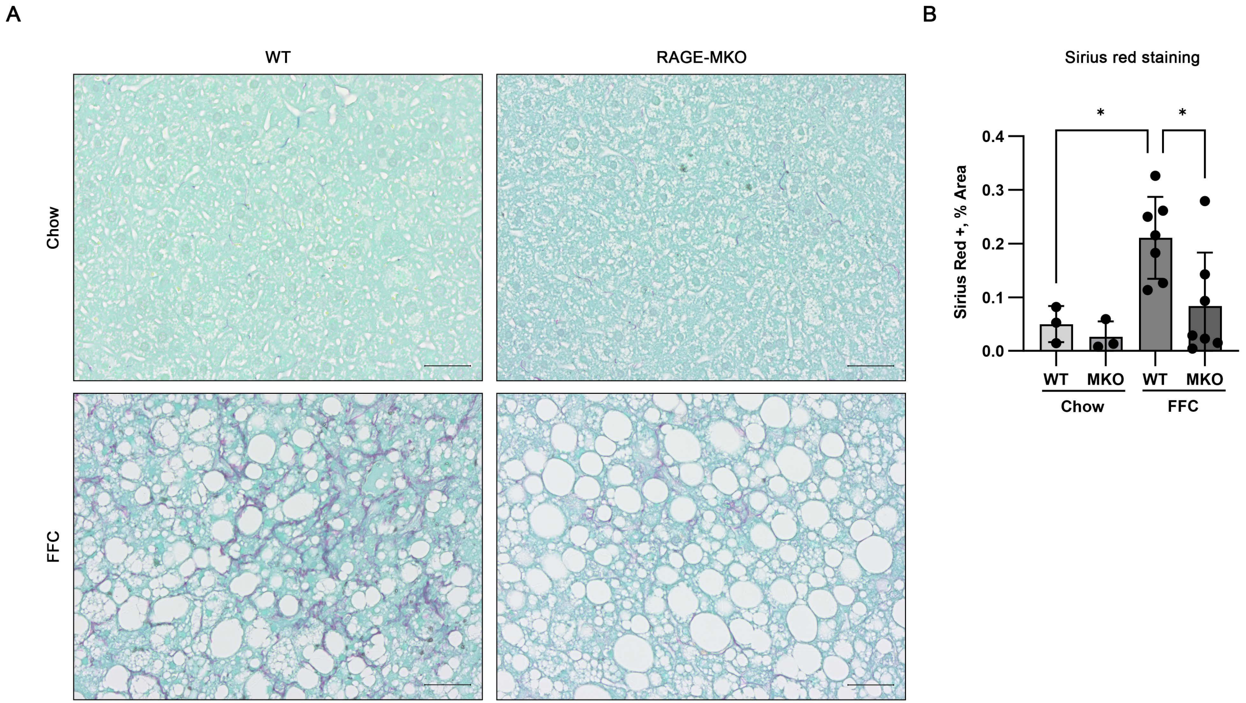

Supplementary Figure 9. Attenuation of FFC diet induced fibrosis with myeloid-specific RAGE knockout.
(A) Representative images of sirius red staining of livers from WT or RAGE-MKO mice fed a chow (n=3 each) and FFC mice (n=7 each) demonstrating collagen staining in red, scale bar=50 μm, and (B) its quantification, *P*<.05. Mann-Whitney test was used for statistical analyses.

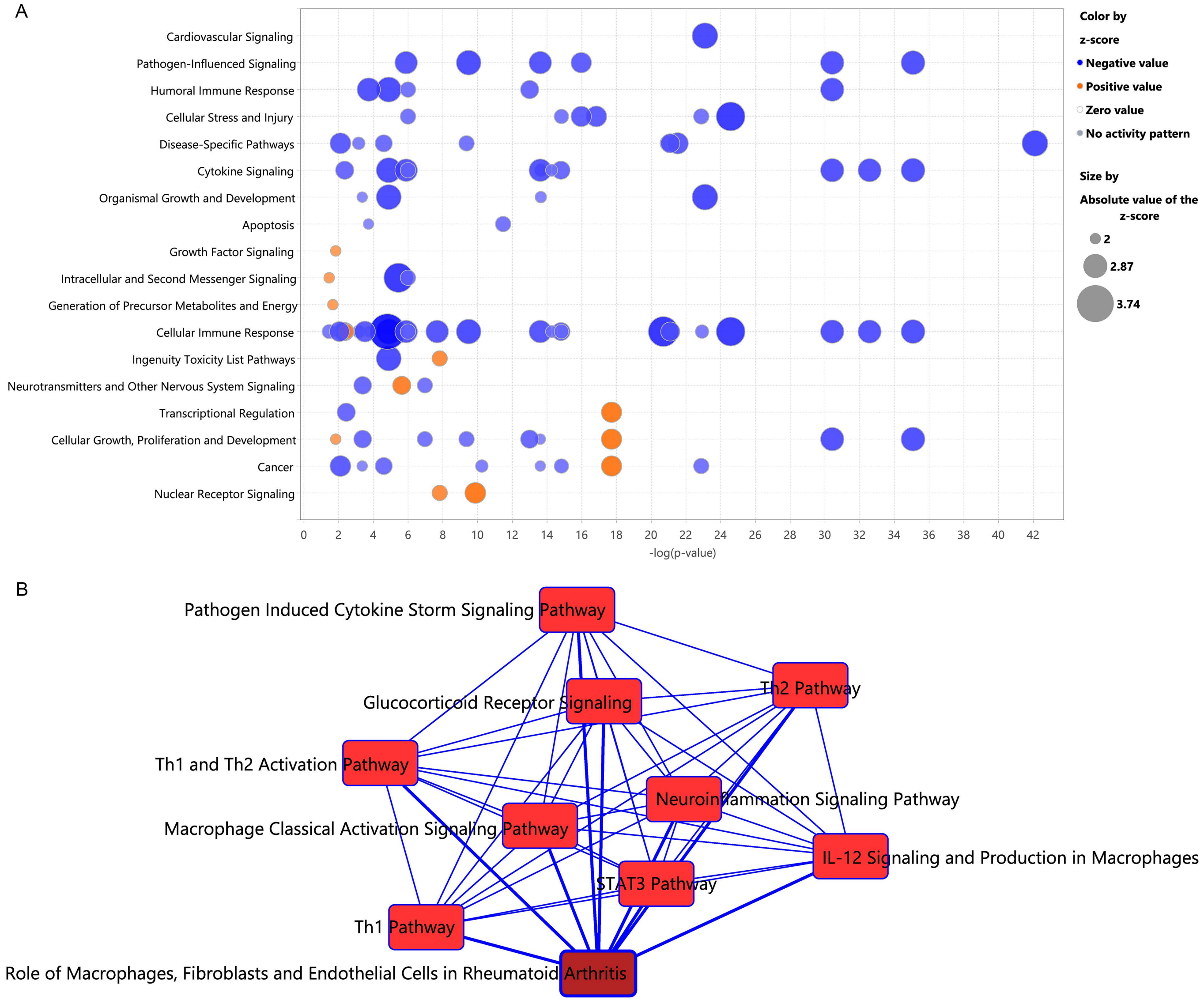

Supplementary Figure 10. Myeloid-specific RAGE knockout attenuates proinflammatory macrophage and T cell signaling. (A) Bubble plot of canonical pathways significantly upregulated (orange) and downregulated (blue) with FFC RAGE-MKO compared to WT mice; (B) Overlapping network of canonical pathways constructed based on shared genes show top macrophage and T cell activation pathways that are consistently attenuated by RAGE-MKO.

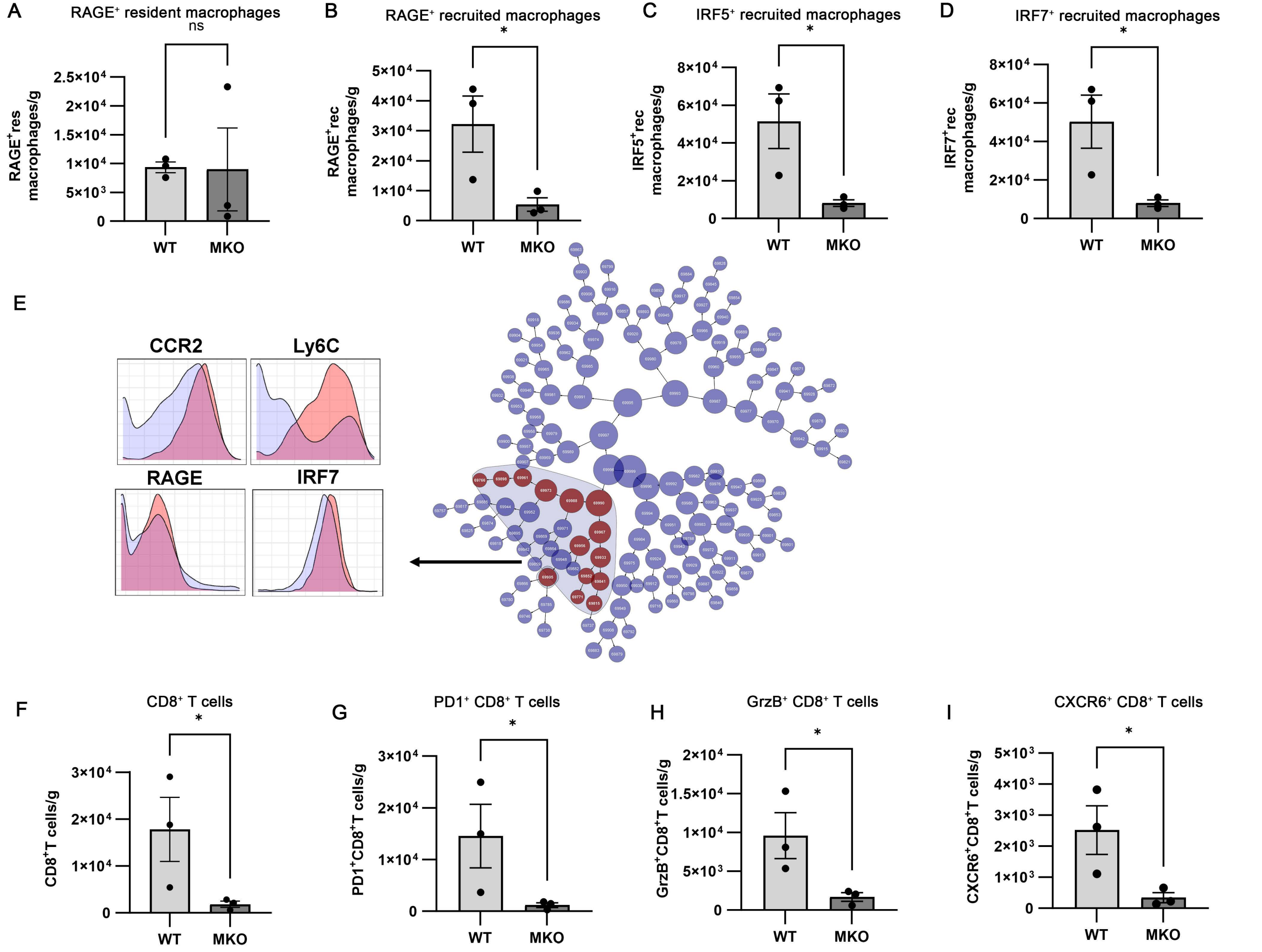

Supplementary Figure 11. Myeloid-specific RAGE knockout attenuates accumulation of RAGE⁺ and proinflammatory macrophage and T cell subsets.

Quantification of abundance of (A) RAGE⁺ resident, (B) RAGE⁺ recruited, (C) IRF5⁺ recruited, and (D) IRF7⁺ recruited macrophages in livers of FFC WT compared to RAGE-MKO (n=3 each, *P*<.05); (E) CITRUS hierarchical tree with shaded groups of macrophage clusters that are less abundant in RAGE-MKO compared to WT mice. The inset show histograms depicting expression intensity of representative markers (orange shaded) compared to other clusters (blue); Quantification of abundance of (F) CD8⁺, (G) PD1⁺CD8⁺, (H) GrzB⁺CD8⁺, and (I) CXCR6⁺CD8⁺ T cells in livers of FFC WT compared to RAGE-MKO (n=3 each, *P*<.05). Cell numbers are presented as cell count per gram of liver. Mann-Whitney test was used for statistical analyses.
